# Comparing child word associations to adult associative norms: Evidence for child-specific associations with a strong priming effect in 3-year-olds

**DOI:** 10.3758/s13428-024-02414-3

**Published:** 2024-06-11

**Authors:** Nadine Fitzpatrick, Caroline Floccia

**Affiliations:** 1 School of Education, Durham University, Durham, UK; 2 School of Psychology, University of Plymouth, Plymouth, UK

**Keywords:** Semantic meaning, Language development, Child, Associative, Taxonomic, Word associations, Stimuli resource

## Abstract

Investigating how infants first establish relationships between words is a necessary step towards understanding how an interconnected network of semantic relationships develops in the adult lexical-semantic system. Stimuli selection for these child studies is critical since words must be both familiar and highly imageable. However, there has been a reliance on adult word association norms to inform stimuli selection in English infant studies to date, as no resource currently exists for child-specific word associations. We present three experiments that explore the strength of word–word relationships in 3-year-olds. Experiment 1 collected children’s word associations (WA) (*N* = 150; female = 84, L1 = British English) and compared them to adult associative norms (Moss & Older, 1996; Nelson et al., 2004 (*Behavior Research Methods, Instruments, & Computers*, 36(3), 402–407)). Experiment 2 replicated WAs from Experiment 1 in an online adaptation of the task (*N* = 24: 13 female, L1 = British English). Both experiments indicated a high proportion of child-specific WAs not represented in adult norms (Moss & Older, 1996; Nelson et al., 2004 (*Behavior Research Methods, Instruments, & Computers*, 36(3), 402–407)). Experiment 3 tested noun–noun WAs from these responses in an online semantic priming study (*N* = 40: 19 female, L1 = British English) and found that association type modulated priming (*F*(2.57, 100.1) = 13.13, *p* <. 0001, generalized η^2^ = .19). This research presents a resource of child-specific imageable noun–noun word pair stimuli suitable for testing young children in word recognition and semantic priming studies.

## Background

Children are sensitive to semantic meaning, in both taxonomic and associative links, by 24 months of age (Arias-Trejo & Plunkett, 2009). This has been measured by comparing whether children attend longer to the second word of a pair of words related in meaning (e.g. *cat*–*dog*) compared to unrelated word combinations (e.g. *cat*–*plate*). However, one striking observation from this research is the absence of a readily available resource of these related words, also referred to as word associations (WAs), in young children to validate the exact relationships between words in early childhood and to inform stimuli selection in research exploring the emergent lexical-semantic system. The lack of such a resource has resulted in a reliance on WAs from the adult literature. To our knowledge, these WAs have not been validated as existing in the lexicons of young children, yet are nonetheless used as stimuli when exploring early semantic development. It could be argued that the demonstration of semantic priming in early childhood (Arias-Trejo & Plunkett, 2009) validates the use of WAs taken from adult norms. However, these effects might not be distributed evenly across all stimuli pairs, and more importantly, the failure to demonstrate priming earlier than 24 months using intermodal preferential looking (Arias-Trejo & Plunkett, 2013) could be due to partially immature word associations due to the selection of stimuli. Thus, it would be of empirical interest to first determine whether WAs are comparable in the adult and the emergent child lexicon. Furthermore, by documenting child-specific WAs, it may highlight some of the first associations that children form and can verbalise, suggesting the primacy of these relationships. Consequently, these early relationships would be more likely to be captured in studies that explore the development of semantic meaning as young as 18 months old (e.g. Delle Luche et al., 2014; Plunkett et al., 2022).

A number of early studies (e.g. Jenkins & Russell, 1960; Koff, 1965; Woodrow & Lowell, 1916) did explore differences between WAs in adults and children, but findings were inconclusive, older children (> 8 years) were tested, and the exact word pairings that children used were not documented and made accessible as a stimulus resource.

Word association tasks have been employed in various areas of psychological research for over a century (Fitzpatrick et al., 2013). In a typical WA task, a participant names or writes the first word they think of in response to a cue word. Exploring WAs can provide insight into the organisation of the mental lexicon and how this organisation affects performance in certain tasks involving memory and verbal response (Comesaña et al., 2014). Through our experience of the world, associative structures form, linking word representations together in the mental lexicon. The shared lexical experience of many people is represented by this associative structure, and the way in which words are associated provides information about the organisation of the mental lexicon (Nelson et al., 2000). When one word readily cues another, the links between the two are believed to have a strong connection in memory (Nelson et al., 2000). This makes the study of WAs a useful tool for investigating meaning and internal representations related to language (De Deyne et al., 2019).

In network models of semantic memory (Collins & Loftus, 1975), concepts are represented in an interconnected network of nodes. Spreading activation occurs between related concepts in such a system so that when one concept is activated, like the cue in a WA task, this activates other nodes related to the concept, such as the responses generated to the cue word. A common opinion is that these WAs represent the links in the network (de Groot, 1989), and by knowing the types of responses (e.g. paradigmatic or syntagmatic), it can reveal the types of links between concepts in semantic memory (Moss & Older, 1996).

However, conceptual links are not the only factor affecting associative strength in words. The frequent co-occurrence of words such as *cat*–*dog* are thought to contribute to the associative strength in addition to their category membership, which means co-ordinates such as *cat*–*horse* would have a lower associative strength to *cat*–*dog,* as the words might belong to the same semantic category, but they do not occur frequently together in everyday language (Moss & Older, 1996).

To date, there have been a large number of adult studies looking at WAs, including studies which document the exact word–word pairs produced by more than one participant (these are discussed further in the next section: Word Association Studies in Adults). In contrast, there have been far fewer child studies looking at WAs and, to the best of our knowledge, none to date have tested British English-speaking children under 4 years old, nor have these studies included a resource of the word–word pairs produced by children that are suitable for use in infant studies. The absence of the exact word–word pairs produced by children in child WA studies to date has resulted in a reliance on adult WA studies that do include word–word pairs, to inform stimuli selection in child studies exploring the development of semantic meaning. For this reason, the next section presents the commonly cited adult WA resources in the child literature, and other large-scale adult WA resources to act as a model for how child-equivalent resources could look.

### Word association studies in adults

Studies investigating infant semantic development often draw stimuli from, and reference the work of, three key adult associative norms studies: the Edinburgh Associative Thesaurus (Kiss et al., 1973), the Birkbeck Word Association Norms (Moss & Older, 1996), and the University of South Florida free association norms (Nelson et al., 2004).

Kiss (1975) and Kiss et al. (1973) collected WAs between 1968 and 1971 from 100 British, 17–22-year-olds for the Edinburgh Associative Thesaurus. There are 8400 cues (taken from Kent & Rosanoff, 1910) with 100 responses per cue. Although this resource is no longer readily available, it has more recently been transformed into an RDF dataset (Resource Description Framework—a model for data interchange on the Web) (Hees et al., 2016). Child studies using this resource to inform stimuli selection include Arias-Trejo and Plunkett (2009, 2013), Chow et al. (2017, 2018), and Mani and Plunkett (2010).

Moss and Older (1996) compiled the Birkbeck Word Association Norms from the associative responses to 2464 words, organised into 14 tests, over 7 years. Participants were between 17 and 45, living in the UK. Each cue word was allocated to 41–50 British English participants, and each participant responded to 50–387 cue words, with some participants completing more than one test session. Child studies using this resource to inform stimuli selection include Arias-Trejo and Plunkett (2009, 2013), Jardak and Byers-Heinlein (2019), Mani and Plunkett (2010), and Styles and Plunkett (2009).

In the University of South Florida free association norms, Nelson et al. (2004) reported the WAs of more than 6000 American adult participants to 5019 cues. A total of 149 participants responded to 100–200 words on average, which generated 72,000 word pairs. The research has been cited 1900+ times and is the most commonly used resource in English (De Deyne et al., 2019), despite data collection starting 40 years before its publication. Child studies using this resource to inform stimuli selection include Chow et al. (2017), Delle Luche et al. (2014), and Jardak and Byers-Heinlein (2019).

A more recent adult study is the English Small World of Words project (SWOW-EN) (De Deyne et al., 2019) which compiled a new English WA dataset, collected between 2011 and 2018. The study tested 12,000 cue words on over 90,000 participants. The sample included over-16-year-olds who were predominantly American English and British English speakers.

Due to inconsistencies found in the methodologies used in a number of influential adult WA studies, Fitzpatrick et al. (2013) devised a WA task to explore differences in WAs, modulated by age. Twin 16-year-olds and twins over 65 years old (*N* = 48 twins per group) were tested. Age-related differences were reported, which the authors suggest might stem from the vocabulary preferences of the two age groups or to changes related to ageing. Consequently, Fitzpatrick et al. (2013) caution against using normed lists such as the South Florida Association Norms (1998) to compare responses of a target population, as it fails to acknowledge the characteristics of a cohort, such as generational differences, which might influence how a group responds. A population-specific list will reflect the characteristics of those tested, and this will enable better identification of differences within and across populations.

Thus, adult studies are sub-optimal for informing stimuli selection for child studies, and so we now turn to the literature on WAs in children to explore how the methodology commonly used in adult WA studies can be adapted for children, particularly to make the WA task accessible for young children who are not old enough to read or write.

### Word association studies in children

There have been far fewer WA studies conducted on children compared to adult studies. Of the more recent child studies (e.g. Comesaña et al., 2014; de La Haye et al., 2003; Macizo, 2000; Zortea & de Salles, 2012), few have tested children under 7 years of age, and few have used an oral methodology. Since the aim of this paper is to develop a resource of imageable, associated word–word pairs that can be used to explore the primacy of semantic meaning, we focus on studies with a well-documented WA methodology that can be accessed by very young children. Many of these studies, however, are much older than more recent work.

The youngest age group tested to date in a WA study seems to be 48–66 months in the WA studies conducted by Newman (1970). Using a ‘continued sentence associations’ methodology, which encourages multiword responses, and a standard WA methodology, Newman found that the former was more successful when testing children at a young age. Unlike adult, single-word WA responses, a common tendency in children of 4–5 years engaged in associative word tasks is to respond with more than one word (see Entwisle, 1964). This offers insight into how to adapt a WA methodology for even younger participants.

An area of particular interest in WA research in children is investigating the occurrence of a developmental shift referred to as the ‘syntagmatic–paradigmatic’ shift (White, 1985). As per definitions used in previous WA studies with children (Sheng et al., 2006; Wojcik & Kandhadai, 2020), a paradigmatic response in a WA task might be defined as a superordinate (e.g. *cat*–*animal*), a subordinate (e.g. *train*–*carriage*), a synonym (e.g. *brush*–*comb*), an antonym (e.g. *night*–*day*), or a category coordinate (e.g. *elephant*–*dog*). A syntagmatic response can be defined as a word which is able to syntactically follow or precede the cue (e.g. *train*–*track*), or which is thematically close (e.g. *bed*–*story*).

Until 6 years of age, children’s responses to a WA task are mostly based on syntagmatic links (Brown & Berko, 1960; Entwisle et al., 1964; Ervin, 1961), but after this age, up until 11 years, children’s responses become more paradigmatic in nature (Newman, 1970).

Paradigmatic responses (e.g. *insect* after *bee*) to a WA task indicate a more developed semantic system, thus are more common in adult associated responses. It is believed that a higher level of cognitive processing is behind this type of response, which involves processes such as conceptual and lexical reorganisation (Nelson, 1977). Thus, as children develop cognitively and linguistically, it is thought that the types of WAs they produce will become more adult-like, and paradigmatic in nature. Paradigmatic knowledge helps structure semantic networks and the retrieval of semantic knowledge, which develops as a child increases their vocabulary (Sheng et al., 2006). However, according to Wojcik and Kandhadai (2020), the assumption that young children only produce syntagmatic responses (e.g. *honey* after *bee*) in a WA task is inaccurate, because taxonomic responses (e.g. *horse* after *dog*) are produced by children, but there is simply a lack of data in the WA literature testing children. In fact, in experiments testing comprehension, sensitivity to syntagmatic and paradigmatic relationships between words has been observed at 24 months (Arias-Trejo & Plunkett, 2013), with some evidence suggesting the existence of paradigmatic relations as young as 6 months (Bergelson & Aslin, 2017).

To explore the developmental trajectory of paradigmatic relations in children, Wojcik and Kandhadai (2020) conducted a WA task on 60 English-speaking 3-8-year-olds (*M*= 4.85, *SD=* 1.27). They also tested a group of adults for comparison (*N*= 60). A total of 65 cue words were used (nouns = 25), and eight order lists were created, 32–33 words in length. Children were grouped as ‘old’ at 6–8 years (*N* = 17) and ‘young’ at 3–5 years (*N* = 43). The authors found clear evidence of paradigmatic responses in ‘young’ children, with a higher proportion of this response type in ‘old’ children, and a higher proportion still in adults.

Much like other recent WA studies testing children, a limitation to this study is the relatively small sample tested (e.g. Cronin, 2002: *N* = 59; Sheng et al., 2006: *N* = 24; Wojcik et al., 2020: *N* = 60). While much larger-scale English WA studies exist in children, many of these were conducted over 50 years ago (Entwisle, 1966). One such study was conducted in 1963 by Koff (1965), who tested 8- to 12-year-olds (*N* = 147) on a list of 51 words to compare children’s associative responses with responses collected in one of the first child studies on WAs (Woodrow & Lowell, 1916, testing children aged 9–12, *N* = 1000). Koff found a significant difference in primary responses in children from 1916 to 1963, but when compared to adult responses given in 1954 (Jenkins & Russell, 1960), there was not a large difference between responses given by children and adults. This differs to Woodrow and Lowell’s (1916) finding of a large discrepancy between children and adults. Koff (1965) concluded that a cumulative effect on WAs can be attributed to changes in culture.

Taken together, it is clear that only a few studies directly elicit free associations from children under the age of 4, and that large-scale WA studies conducted on English-speaking children are already very old. Whether the associated responses of English-speaking adults and children are similar (Koff, 1965) or very different (Woodrow & Lowell, 1916) remains inconclusive.

## Proposed research and rationale

The WA literature reviewed indicates that caution must be taken not to generalise findings from normative studies across different populations, as these will have their own associative norms (Nelson et al., 2004). Word associations are likely to be modulated by age (Fitzpatrick et al., 2013), and if associations stem from our experience of the world and our exposure to linguistic input, this will inevitably differ according to the stage of a child’s linguistic development. Common relationships between words in young children might be missed if relying on predetermined relations (Wojcik & Kandhadai, 2020) which do not derive from the population of interest. Due to a lack of studies documenting very young English-speaking children’s WAs, and no studies to our knowledge testing under the age of 4, it remains to be seen what some of these early word–word relationships are, and whether they mirror adult associative norms (Arias-Trejo & Plunkett, 2009), which are the source of stimulus selection in many infant studies exploring early word–word relationships.

To date, many child studies have relied on adult associative norms for their stimulus selection, yet these norms do not prioritise highly imageable word pairs, which is imperative when testing young children. Therefore, the aim of this research is to develop a task whose focus it is to document common noun–noun WAs in the lexicon at as young an age as possible (Experiment 1). Then the aim is to replicate these word–word connections through a second study (Experiment 2) and to determine whether these connections are equally strong in a receptive, semantic priming study (Experiment 3). Together this will provide evidence that these words are connected in the lexicons of young children receptively as well as productively and can therefore be reliably consulted as a stimulus resource for future studies investigating the development of lexical-semantic networks in English-speaking infants.

## Experiment 1

Since few studies have collected WAs in very young children, and no study to our knowledge has tested children under the age of 4, we based our method on Newman’s (1970) WA methodology which encourages more than one attempt to respond to a cue word (see Newman, 1970, Experiment 2), acknowledges multi-word responses, uses a reduced number of cue words compared to other experiments, and has an oral mode of delivery. All of these elements likely make it a more accessible WA method when testing young children under 4, who are not yet able to read or write, and who have not yet been reliably tested on such a task to know how we might optimise the process for young children with limited language. We hypothesised that by using a methodology as outlined above, particularly one that allows for more than once response, it may allow this young age group to use repetition or rhyme as a tactic to processing the cue (Palermo & Jenkins, 1964) while learning how to respond correctly to the task; in addition, it better frames the task as a ‘word game’ (see Palermo & Jenkins, 1964, 1966; Palermo, 1971), which might help with engagement, which is a concern when testing young children.

The WA task was administered quite differently to previous studies: the at-home format (Experiment 1) saw the parent act as experimenter, whereas the online format (Experiment 2) used puppets to model the task and take on the role of experimenter. These decisions were taken to accommodate the young age of participants and to allow testing to continue during the UK national lockdown at the start of the COVID-19 global pandemic.

### Method

#### Participants

A total of 150 participants^1^ (female = 84, male = 66) completed the study. Of those, 140 were recruited from the BabyLab database and its corresponding Facebook page, and the remainder were recruited from other Baby Labs. Participants were divided into seven 2-month age bins, i.e. 34–35 (*N* = 23), 36–37 (*N* = 22), 38–39 (*N* = 21), 40–41 (*N* = 20), 42–43 (*N* = 21), 44–45 (*N* = 23), and 46–47 (*N* = 20), to explore WA production across a child’s third year of life^2^. Participants were considered ineligible for the study if known to speak more than one language, or if diagnosed with a developmental or language delay. These eligibility criteria apply to Experiments 1–3.

#### Materials

One hundred highly imageable, concrete nouns were selected from nine categories (e.g. animals, toys, clothes) that are known by at least 60% of 18-month-olds according to the Oxford Communicative Development Inventory (CDI; Hamilton et al., 2000) and UK CDI (UK-CDI Database, 2016). The full list of words can be found in Appendix Tables 3. Ten lists of 10 words were created, ensuring each category was represented in each list. Two pseudo-randomised orders were created for each of the 10 wordlists to avoid effects of cue order. Care was taken to avoid consecutive words being associatively related or appearing from the same category. Words sharing initial word onset were not presented consecutively.

#### Procedure

After we received ethical approval from the university’s ethics committee, participants meeting the inclusion criteria were contacted via the BabyLab database or Facebook page. An email invitation including a participant information sheet outlining the procedure, data handling, and a consent form were sent. Written consent was obtained from the parents. At the end of the process, a final debriefing email was sent out thanking the family for their participation in the study with a digital certificate and £5 voucher code attached. Experiments 1–3 all followed this procedure.

Next, interested families were sent an email with the task instructions and one of the 10 wordlists. On receipt of this, parents were asked to request replacement words if the words were unfamiliar to their child. We used parental report to determine a child’s comprehension of each word, in line with the procedure for administering the MacArthur-Bates CDI-III (CDI, Fenson et al., 2007, lexical component only).

Parents were instructed to follow the script (see Appendix Tables 4) as closely as possible and to elicit three responses per cue where possible. Parents were asked to use the cue word when encouraging each of the child’s three responses to a word. It was emphasised that the task should be enjoyable and that the parent should move on to the next word if their child had difficulty responding. Parents were instructed to record their child’s responses in the order they were given, in a table provided (see Appendix Tables 4). The full utterance of a response was requested, with instruction to indicate whether the child was naming objects in the immediate environment.

Parents returned the completed task by email to the experimenter. The responses were checked, and parents were contacted to provide further information about ambiguous responses, especially if seemingly random responses might have related to something in the immediate environment. Previous research on free associations in children (Palermo, 1971, 1964) has shown this to be common when young participants are unable to produce a response.

#### Pilot study

A pilot study was run on children between 24 and 60 months (*N* = 14), but 24–30-month-olds were not always successful in understanding the task, with some unable to complete it at all. This prompted a change in the minimum age from 24 months to 34 months. Due to availability of resources and a refocusing of the research aims, the upper age limit was set to 47 months to focus on WAs in the third year of life.

### Results

#### Data processing

Data were pre-processed as follows: spelling errors were corrected; nouns were prioritised when a word belonged to multiple word classes; contextual information provided by parents was noted in brackets to assist coding; and missing responses were marked as ‘NO RESPONSE’.

#### Coding for response type

Different response types were identified by analysing the data collected in the pilot study, leading to a set of 10 categories: Category 0 = no response; 1 = related; 2 = unique relationship to child; 3 = connected to a previous response; 4 = related in a wider sense; 5 = repetition of cue; 6 = naming something in immediate environment; 7 = unrelated; 8 = rhyme (including clang responses); 9 = sounding out (e.g. APPLE – ‘a’ for apple); 10 = sound or action (see Appendix Tables 5 for a more detailed description with examples). Related responses were tagged as paradigmatic, syntagmatic, or both. Definitions used in previous WA studies with children (Sheng et al., 2006; Wojcik & Kandhadai, 2020) and as mentioned previously^3^ were adopted.

Participant responses were coded by the lead researcher, with a junior researcher coding a subset (10%) of the data. Rater agreement of category coding was 91% with a Cohen’s κ of 0.62 which demonstrates substantial agreement (Landis & Koch, 1977). Paradigmatic/syntagmatic coding agreement was 93%, with a Cohen’s κ of 0.92, demonstrating near perfect agreement.

#### Associative strength analysis

The likelihood of a cue word producing a particular response in a WA task (e.g. cat -> dog) can be indexed using a measure of forward strength (FSG, Nelson et al., 2000). This is calculated by dividing the number of participants producing a particular response to a cue (*P*) by the total number in the group responding to a given cue (*G*): FSG = P / G.

To calculate *P*, the data were first grouped. For example, responses were grouped for a repeated entry, and for the plural and singular forms of a noun (see Entwisle, 1966). In multi-word utterances containing a noun, the noun was the focus (in line with the aim of this study).

The FSG was calculated for every response produced by two or more participants following the procedure used by Nelson et al. (2000). This was done to generate a proportion which could be compared to other datasets looking at FSG in WAs (Moss & Older, 1996; Nelson et al., 1998).

#### Descriptive statistics

A total of 4512 responses were collected from 150 3-year-olds completing the WA task. After subtracting responses categorised as ‘no response’ (i.e. Category 0, *N* = 908), a total of 3603 responses remained. This produced an average of 24 responses out of a possible 30 (three attempts for each of the 10 cue words, SD = 6.14). Considering first responses only, out of a possible 1500 responses (150 participants, each with 10 cue words), 1454 responses remained after subtracting ‘no responses’ (*N* = 46). The mean response rate was 9.69 (SD = 0.91).

**Fig. 1:**
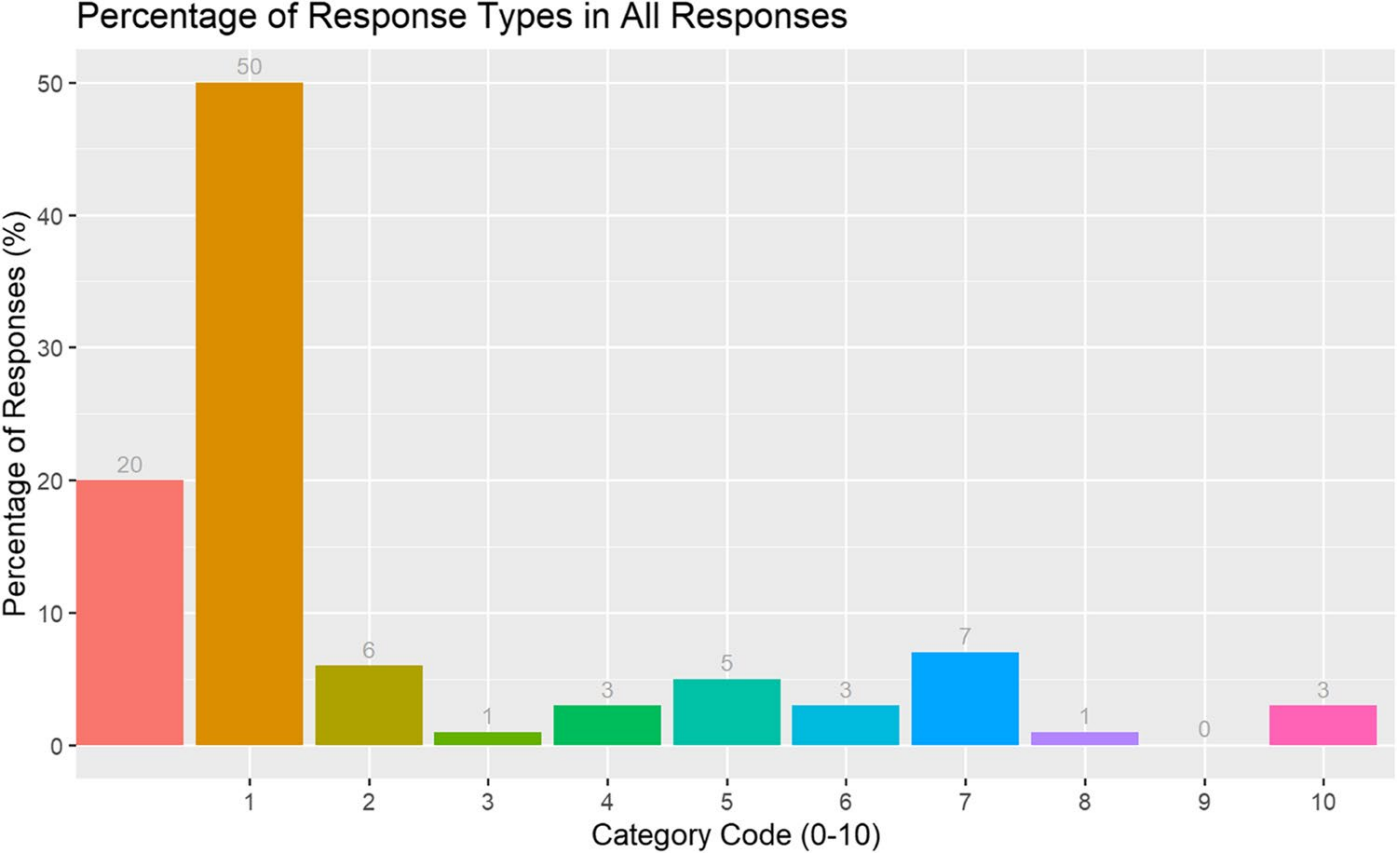
Experiment 1. The percentage of WA responses (all responses) by response category: Category 0 = no response, 1 = related, 2= unique relationship to child, 3 = connected to a previous response, 4 = related in a wider sense, 5 = repetition of cue, 6 = naming something in immediate environment, 7 = unrelated, 8 = rhyme, 9 = sounding out (e.g. APPLE – ‘a’ for apple), 10 = sound or action

We then calculated the percentage of all responses per category type (see Fig. 1). Most 3-year-olds’ responses were related (i.e. Category 1), rather than any other type of response.

By organising responses into no response (Category 0) and collapsing categories representing a related response (Categories 1, 2, 4, 10) and responses which are not related (Categories 3, 5, 6, 7, 8, 9), Table 1 illustrates the distribution of all responses, as a percentage and as a raw value.

**Table 1:** Experiment 1. Percentage of all responses by relatedness of response type

	Number of responses	Percentage
No response given	908	20%
Related response	2807	62%
Unrelated response	797	18%

**Fig. 2:**
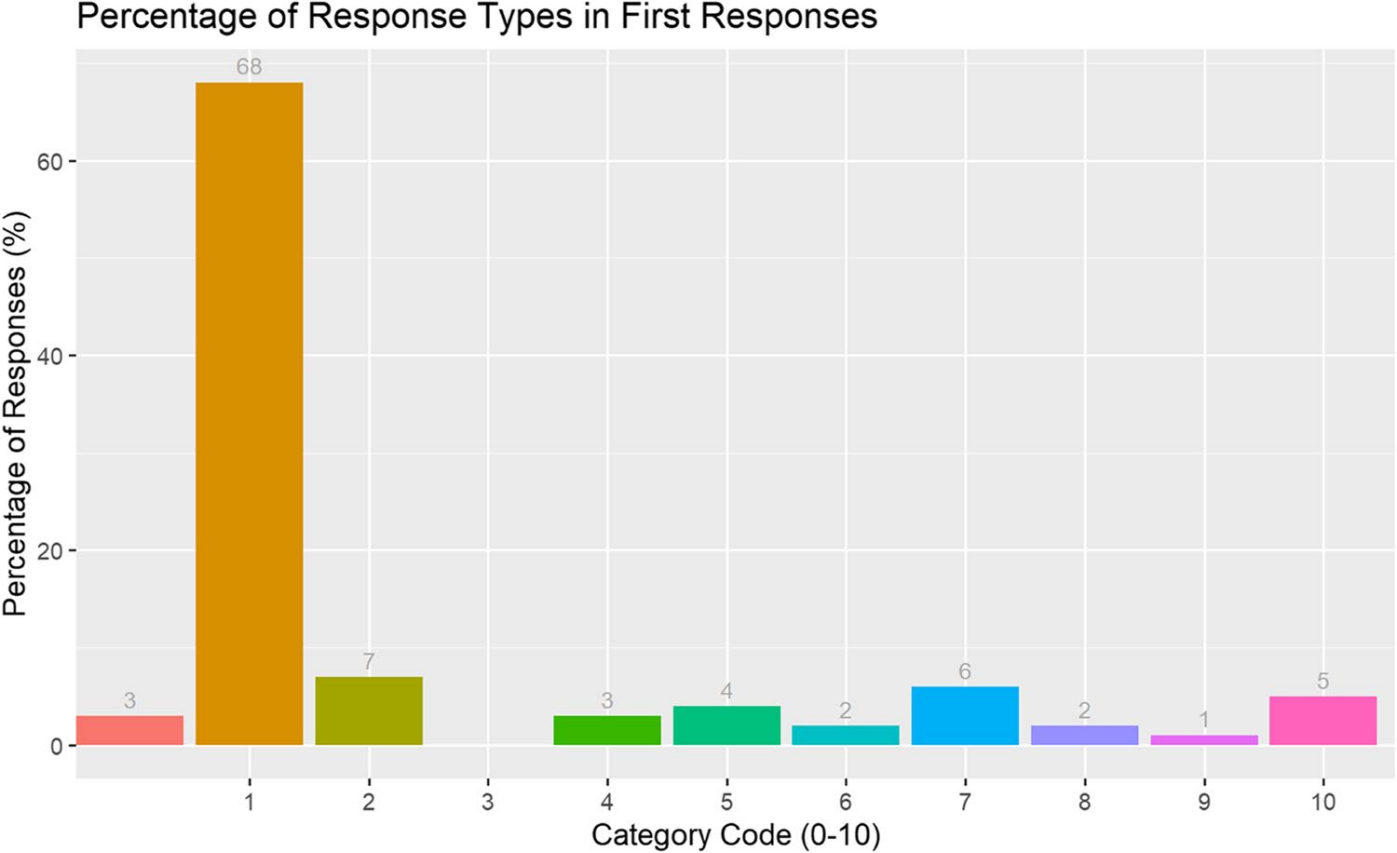
Experiment 1. The percentage of WA responses (first responses only) by response category

Next, we calculated the percentage of responses per category type for first responses only (see Fig. 2).

By splitting the data in this way, we see a higher percentage of related responses (Category 1 = 68%) and lower instance of no responses (Category 0= 3%). Due to this observation and since not all children gave three responses to every cue word, we focus henceforth on first responses only for inferential analysis, but we have retained related responses from second and third responses in the Appendices to document exact cue–target word combinations.

Given that some participants did not provide a response for each of the 10 cue words, a proportional score of related responses was calculated for each participant. This was the number of related responses divided by the total number of responses (minus no responses). The overall mean proportion of related responses was 0.85 (SD= 0.21). We ran a type III ANOVA on participants’ proportion of related responses with gender and age bin as fixed factors. There were no significant differences between the proportion of related responses by gender and age, and no interactions between the variables (*p*s > 0.1).

Related first responses were categorised as paradigmatic, syntagmatic, or both. Following Wojcik et al.’s (2020) method of calculation, responses classified as paradigmatic or both were combined. A total of 25.2% of responses were paradigmatic (or both), and 74.8% of responses were syntagmatic. We ran a type III ANOVA on participants’ proportion of paradigmatic responses with gender and age bin as fixed factors. There were no significant differences between the proportion of paradigmatic responses by gender and age, and no interactions between the variables (*p*s > 0.1).

#### Associative strength

Related responses given by two or more children to each of the 100 cue words were processed to calculate their forward word association strength (FSG) (Nelson et al., 2000). Focussing on first responses only^4^, a total of 188 responses had two or more participants producing the same response for a cue word, with 96 of the cue words represented in these responses. The full list of cue words (organised alphabetically) with two or more of the same response and their associative strengths (*M* = 0.20, range = 0.11 to 0.69) can be found in Appendix Tables 7.

Since one aim of this research was to look at the most common imageable noun–noun associated word pairs in 3-year-olds, we extracted noun–noun word pairs to create a stimulus resource bank (see Appendix Tables 8**)**. Of the 188 responses shared by two or more children, 115 of these were noun–noun word pairs.

To determine whether the most common WAs in our sample of 3-year-olds are unique to this age group, we then compared the FSG from the adult literature for the same word combinations. Of the 188 related word–word combinations produced as first responses by two or more of the 150 3-year-olds in this study, 30 were not characterised in either the Birkbeck or the South Florida norms (though the cue was used); 13 were not used as a cue in the Birkbeck norms, nor documented as an associated response in the South Florida norms; two were not documented as an associated response in the Birkbeck norms, nor used as a cue in the South Florida norms; and four were not used as a cue in either study, resulting in a total of 49 word pairs found in children’s responses, without a value of associated strength in adults. This missing data correspond to a total of 26% of associated responses found in 3-year-olds that are not reflected in adult associative norms^5^.

The resulting 139 word pairs which are represented in the adult data were analysed. Where there was an associative strength available in the two adult studies used for comparison (Moss & Older, 1996; Nelson et al., 1998), the mean was taken, but where only one value was available, this was taken to represent FSG in adults. The 139 word pairs can be seen in Appendix Tables 9.

A paired *t*-test was run to determine any difference between the associative strength between word pairs in children and adults. There was a significant difference in the FSG between age groups, *t*(138) = 4.58, *p* < .001, 95% CI [0.04, 0.10], indicating stronger associative strength between word pairs in children (*M* = 0.21, range = 0.11–0.69) compared to adults (*M* = 0.14, range = 0.01–0.76). There was a significant, weak positive correlation between the two groups, *r*(137) = .22, *p* = .01, 95% CI [0.05, 0.37]. This shows a tendency for strongly associated word pairs in adults to be strongly associated in children too.

Some of the WAs with the highest FSG in the data are not replicated in the adult literature, so while no comparison can be made statistically, these may represent novel WAs in 3-year-olds that warrant further testing. These word combinations are displayed in Appendix Tables 10.

### Discussion of Experiment 1

Experiment 1 tested whether children as young as 3 years old could successfully complete a WA task and sought to compare any recurring responses in children to those found in adult norms using forward associative strength as the metric of comparison. There was strong evidence that children between 34 and 47 months can produce associated responses in a repeated free association task. In fact, 3-year-olds produced related responses for the majority of their responses (62%). This establishes that 3-year-olds can successfully complete a WA task and produce some of the same responses as their peers, rather than just idiosyncratic responses.

A large number of associated first responses were produced by two or more 3-year-olds; however, only 139 of these associatively related pairs could be found in adult associative norms. In other words, 26% of related responses given by two or more children are not found in adult norms, and this includes some of the word pairs with the strongest associative strength found in the child data. This might provide a glimpse into the shared experiences of 3-year-old children, which is represented in their lexical-semantic structure at this age. However, these findings would need to be replicated to draw any inference about the probability that a particular cue will elicit an expected associated response in a 3-year-old. This will be addressed in Experiment 2.

Most (74.8%) of the related responses given by 34–47-month-olds were syntagmatic, and there was no effect of age on the rate of paradigmatic responses in the third year of life. The tendency for 3-year-olds to produce syntagmatic responses in a language production task is in line with the idea that a shift to paradigmatic responses in a WA task occurs later, at 6 years of age.

The findings from this study suggest that adults and children converge in the likelihood that certain cue words will elicit the same associative responses; however, this is only true for some word pairs. A direct comparison is difficult to make between the associative strengths found in children and adults, as not much is known about the variables affecting WA behaviour (Fitzpatrick et al., 2013).

A potential explanation for why the associative strength between word pairs might be higher in children compared to adults is due to 3-year-olds having smaller vocabularies, and therefore, the connections that exist between words in their mental lexicons could be stronger, as they are fewer in number.

## Experiment 2

Findings from Experiment 1 validated the use of a free association task on 3-year-olds when the task is administered by a parent. However, having the parent act as ‘experimenter’ inevitably calls into question the validity of the task’s administration, and indeed informal correspondence with participants indicated that there were some deviations from the delivery of the task when performed by different families in their unique home contexts. While this may not directly influence the types of responses a child gives, it warrants a replication study to confirm that when a parent administers the task at home, the types of WAs that a 3-year-old produces in this context are the same types of responses that would be given in a more controlled setting. This potential confound has prompted an adaptation of the original methodology into an online format.

The online WA task did not require the parent to act as the experimenter, but instead used pre-recorded videos of puppets to describe and demonstrate the task. A participant’s responses were recorded for off-line coding, and the more engaging format sought to retain the child’s focus. A further impetus to test online was the inability to test face-to-face due to the global pandemic.

In Experiment 2, we asked whether the WAs produced by 3-year-olds in the parent-administered version of the task could be replicated in another modality, that is, in an online format. To what extent the modality influenced the responses was addressed, as well as examining whether word pairs found in Experiment 1 re-occurred in this online modality, and whether their associative strength was replicated.

The task remained very similar in its design through its remote administration, for instance, by using the same cue words, and with 10 cue words and three responses encouraged for each cue word. However, a homogeneous delivery of the task was better achieved by controlling how the task was explained and how responses were recorded.

We adjusted the age range for Experiment 2 to 36–39 months due to restrictions on time and resources. This specific age range was chosen to maintain a focus on very young children (i.e. at the younger end of a child’s third year of life). From the 10 lists of cue words in Experiment 1, cue words eliciting the WAs with high FSG were selected to create two new lists with 10 words per list for Experiment 2.

We predicted that overall, there would be a replication of the WAs with strongest associative strength in 3-year-olds in the modified online modality. However, due to a high idiosyncratic response rate in young children (Wojcik & Kandhadai, 2020), the strength of the WAs and specific word pairings may differ for Experiment 2. If the parent acting as the ‘experimenter’ was a confounding factor in Experiment 1, then we expected a marked difference in the types of the responses produced by participants (e.g. fewer related responses). Equally, if the online modality made the task more engaging, we expected to see a reduction in the naming of objects in the immediate environment and potentially a greater proportion of related responses.

### Method

#### Participants

Monolingual English-speaking toddlers were recruited from the BabyLab database and its social media platform pages (*N* = 24: 13 female, 11 male). The mean age of participants was 37.64 months. Participants were divided into two age bins, 36–37 months and 38–39 months (±15 days), with 12 children in each age bin. CDI III scores (Fenson et al., 2007, lexical component only) were collected from participants, but only approximately a third of parents completed this part of the task (*N* = 7, *M* = 79.43/99, *SD* = 13.62).

### Materials

#### Stimuli

Twenty of the cue words from Experiment 1 which generated a WA with high FSG in Experiment 1 were selected and organised into two new lists for Experiment 2. List 1 comprised chair, bed, tooth, finger, key, sock, bowl, head, park, and bath. List 2 comprised table, teddy, brush, hand, door, foot, cereal, hair, swing, and towel.

#### Audio and video recordings

The script used by parents in Experiment 1 was adapted for use online. The task explanation and examples were delivered by two puppets, with greater exemplification (i.e. more than one example to demonstrate the task) to aid conceptual understanding of the task. Video recordings were made of the puppets explaining and demonstrating the task by two female, junior researchers, all directed and overseen by the author. Great effort was taken to make the instructional delivery engaging by using child-directed speech. In addition to the main explanatory video, short motivational clips were recorded of the puppets encouraging participation and praising a participant’s effort. Cue words were recorded auditorily by the same junior researchers and presented without the puppets on screen to minimise distractions.

#### Procedure

Parents indicated the day and time they would complete the online experiment, and a unique link was generated for the Gorilla Experiment Builder platform (www.gorilla.sc, Anwyl-Irvine et al., 2019), with further instructions on the procedure. Clicking on the link took the participants through a series of tasks, in the following order: study overview screen; participant eligibility questionnaire; consent form; audio and video test screen with equipment eligibility questionnaire; participant and parent/carer demographic questionnaire; word checklist; CDI III (lexis component only); debrief (see https://app.gorilla.sc/openmaterials/764752 for the full procedure). An experimenter was available for questions and troubleshooting during the time the participant attempted the task.

For the WA task, a video was played of a demonstration of the task by two puppets. The puppets gave examples of WAs (using words not in the stimulus list) with an emphasis on the need to say the first thing that came to mind as quickly as possible.

Following the puppets’ instructions, a cue word was played while an abstract, visual attention getter appeared on screen to maintain the child’s attention to the task/on screen. The cue word was presented once with on-screen instructions for the parent to support the child in producing three responses per cue word. An audio recording of the child and parent was made through the participant’s device. Due to the remote nature of testing, this procedure could not be fully controlled, and there is a chance that the parent did not use the cue word to encourage second and third responses. The result of this is the chance of chained responses. However, we included a category code to capture any instance of this (3 = connected to a previous response).

When clicking on ‘Next’ for a subsequent cue word, a video of the puppets praised the child’s attempt, and three text fields appeared for the parent to type the child’s responses in, in the order given**.** This feature was added in case of an error with the audio recording, or a difficulty understanding the child’s speech, and to analyse how parents record their child’s responses. Refer to Appendix Fig. 8 to see how the experiment looked for the parent and child.

On every trial, the parent was able to determine when the child was ready to progress to the next word in the list by clicking on a ‘Next’ button. This allowed for individual differences in the time needed to produce up to three related words. It was made clear to parents to move on if a child could not think of three responses or if a child became disengaged. Additionally, an ‘Exit’ button was present on every screen to end the task if the child did not want to continue. After five words had been presented in this vein, a video of the puppets demonstrated the task again with a non-cue word. The final five words were then tested. Finally, the parent completed a digitalised version of the CDI III (lexis component only)^6^ before a final debrief questionnaire asking for any questions or comments relating to their experience of the task.

#### Piloting

Various iterations of the Gorilla experiment were trialled on junior researchers and children to ensure that the sequence of tasks was optimal and that the instructions for the parent were straightforward and unambiguous. Piloting resulted in the following modifications to the procedure: a hardware eligibility check; optimisation of audio and video for varying bandwidths; restriction of the task for use with the Google Chrome browser; and various modifications to task instructions.

#### Data processing and analysis

Audio responses were transcribed and compared to parental reports of their child’s responses. The rate of agreement between the audio transcription and parental report was 92%, providing sufficient evidence to use parental responses for further analysis. The 8% discrepancy in recorded responses was likely due to the audio recording not capturing all responses (i.e. a child continued talking when the recording stopped), parents not accurately recording/not remembering to record all words uttered, or parents not acknowledging all responses as valid.

Reponses were grouped and categorised (0–10, see Appendix Table 5) by two independent coders, as previously outlined in Experiment 1. The agreement between raters was ‘perfect’ with 100% agreement (Cohen’s kappa). This high level of agreement indicates that the categories were being applied consistently when different coders categorised responses.

Rater agreement for paradigmatic/syntagmatic coding was ‘almost perfect’ at 96%, κ = 0.82.

### Results

#### Descriptive statistics

A total of 593 responses were recorded as related or unrelated out of a possible 720 responses. Remaining responses were ‘no responses’ (*N* = 127). Based on a participant producing up to three responses for each of the 10 cue words, an individual participant produced an average of 24.71 responses (*SD =* 5.80).

**Fig. 3:**
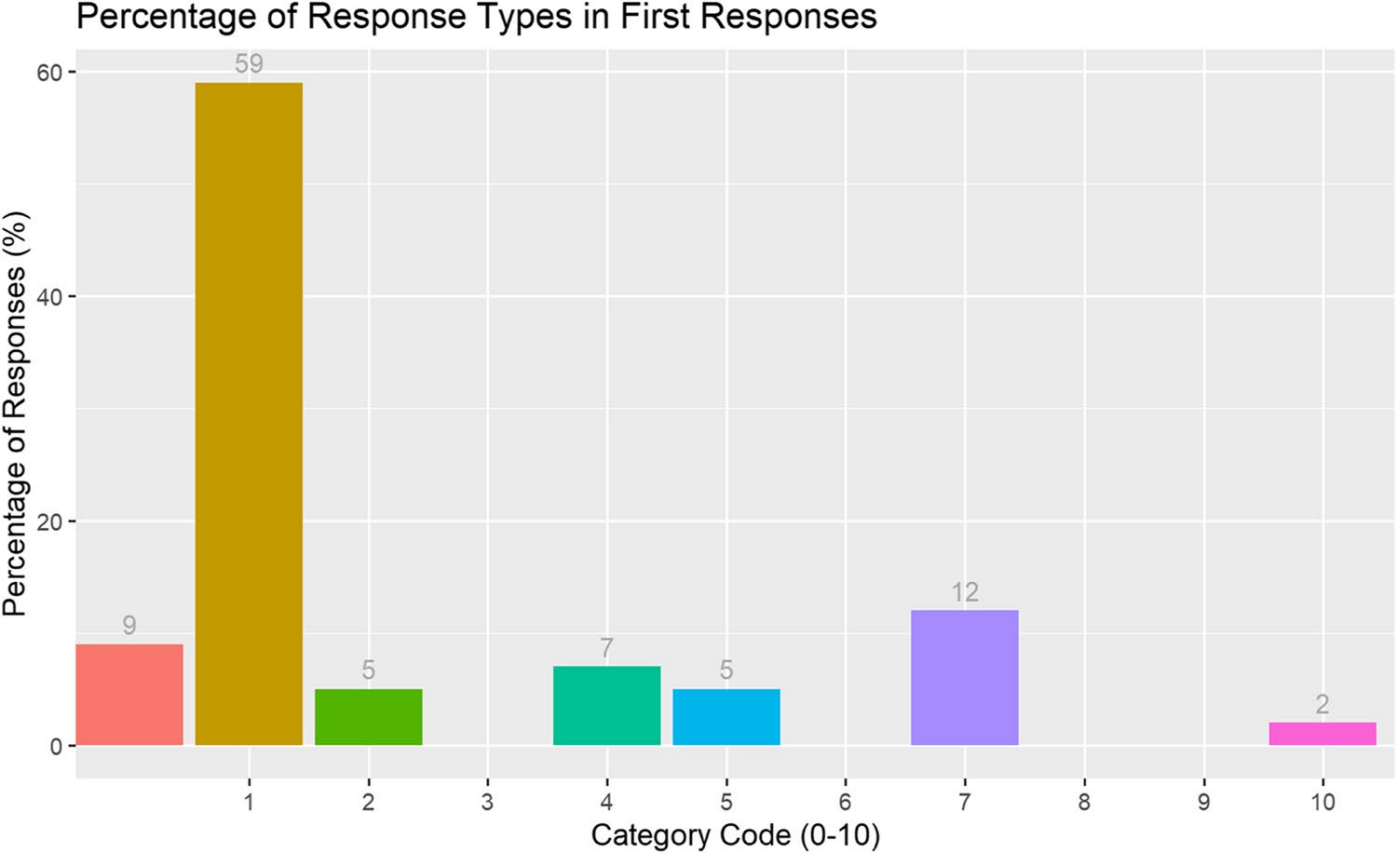
Experiment 2. Percentage of first responses by response category in the online WA task

Considering first responses only, out of a possible 240 responses (24 participants, each with 10 cue words), 218 responses remained after subtracting ‘no responses’ (*N* = 22). Mean response rate was 9.01 (*SD* = 1.61). Figure 3 shows the percentage of first responses by response type.

Category 1 (Related) responses were most prominent (59%), followed by Category 7 (random responses, 12%), then Category 0 (no responses, 9%).

Organising responses into ‘no responses’ (Category 0), a related response (Categories 1, 2, 4, 10) and an unrelated response (Categories 3, 5, 6, 7, 8, 9), Table 2 illustrates the distribution between the three main response types as a percentage and as raw values.

**Table 2:** Experiment 2. First responses by relatedness of response in the online WA task

	Number of responses	Percentage
No response given	22	9%
Related response	178	74%
Unrelated response	40	17%

As per Experiment 1, we calculated a proportional score of related responses (first responses only) for each participant. The overall mean proportion of related responses was 0.82 (SD = 0.19). We ran a type III ANOVA on participants’ proportion of related responses with gender and age bin as fixed factors. There were no significant differences between the proportion of related responses by gender and age, and no interactions between variables (*p*s > .05).

A total of 72% of first related responses^7^ were syntagmatic, and 28% were paradigmatic (or both).

We ran a type III ANOVA on participants’ proportion of paradigmatic responses for first responses with gender and age bin as fixed factors. There were no significant differences between the proportion of paradigmatic responses by gender and age, and no interactions between variables (*p*s > 0.1).

Taking age as a continuous variable, there was a weak negative correlation between the proportion of paradigmatic responses in first responses as age increased, though this was not significant, *r*(22) = −.20, *p* = .36 , 95% CI [−0.56, 0.22]. Together this indicates that 3-year-olds predominantly produce related responses that are syntagmatic, and this is not modulated by age (between 36 and 39 months) or gender.

#### Associative strength

Responses were pre-processed and organised as per Experiment 1. When the same response to a cue word was generated by two or more participants, its associative strength was calculated (Nelson et al., 2000). Considering first responses only^8^, 25 responses were given by two or more participants with 18 of the 20 cue words represented in these word combinations. The list of first response word combinations shared by 2+ children can be found with their corresponding associative strengths (*M* = 0.22, range = 0.17 to 0.42) in Appendix Table 12.

The corresponding associative strength for the related responses given as first responses was then extracted from adult associative norms (Moss & Older, 1996; Nelson et al., 1998) and compared to the child data (see Appendix Table 12). Associative strength was averaged across the two adult studies where possible; otherwise, an available value from one of the studies was taken to represent the associative strength in adults overall.

Seventeen of the 25 associative pairs found in the online free association task were present in the adult associative norms. Eight of the 25 related word pairs found in children’s responses did not have a value of associated strength in adults: three associated word pairs were not characterised in either the Birkbeck or the South Florida norms (though the cue was used); four were not used as a cue in the Birkbeck norms nor documented as an associated response in the South Florida norms; and one was not used as a cue in either study. This corresponds to 32%^9^ of associated responses found in 3-year-olds that is not reflected in adult associative norms.

The associative strengths of related responses in children from the eight cue–response pairs not present in adult norms (*M* = 0.21, range = 0.17–0.33) were compared to the associative strengths of the 17 cue–response pairs present in children and in adult norms (*M* = 0.23, range = 0.17–0.42). There was no significant difference in associative strengths, *t*(23) = −0.72, *p* = .48, 95% CI [−0.04, 0.08], between cue–response word pairs in children only and for pairs found in children and in adult associative norms.

The 17 word pairs which were represented in the child and adult data were analysed further. A *t*-test was run to determine any difference in word associative strength in children and adults. There was no difference in the associative strength between words in the two groups, *t*(32) = 0.87, *p* = .39, 95% CI [−0.05, 0.14], though the associative strength was slightly higher in children (*M* = 0.22, range = 0.17–0.42) than in adults (*M*= 0.19, range = 0.041–0.638). There was no significant correlation between the two groups, *r*(15) = .23, *p* = .38, 95% CI [−0.28, 0.64], despite a weak positive tendency. Associative strength seems to be comparable in adults and children and there is some indication that this could correlate positively: word pairs with high associative strength in adults are also strong in children.

As with Experiment 1, imageable noun–noun combinations with the highest forward associative strength were identified (*N*= 34) and are displayed in Appendix Table 13. These represent the strongest, imageable associated word pairs from the online WA task in 36-39-month-olds (first responses in bold, *N*= 9).

### Comparing experimental modalities: Parental vs. online

In the following section, we compare the two experimental modalities: at home with a parent/carer as the experimenter (Experiment 1) and online, at home with a puppet as the experimenter (Experiment 2), whilst acknowledging that Experiment 2 only tests a subset of the stimulus words (*N*= 20) compared to the stimuli used in Experiment 1 (*N*= 100).

#### Descriptive statistics

There was no difference in response rate between the two experimental modalities, *t*(172) = .44, *p* = .66, 95% CI [0.53, 0.83], which indicates that 3-year-olds approached and responded to the WA task equally when it was performed by a parent in the home, and when demonstrated by a puppet online.

With regards to response type, the pattern of findings in the online WA task clearly mimics the findings in the parentally-administered version of the task. The online experiment replicates the finding of a large proportion of related responses to a cue word, as found when the WA task was administered in the home. This is especially true for the percentage of Category 1 first responses (online- 59%; at-home- 68%), and the overall proportion of related first responses (Experiment 1: *M =* 0.85, *SD*= 0.21; Experiment 2: *M =* 0.82, *SD*= 0.19). Category 0 first responses (online- 9%; at-home- 3%) were also proportionally comparable.

No effect of gender or age on relatedness of response was found in either modality. In both modalities, syntagmatic responses occurred more frequently than paradigmatic responses. The rate of paradigmatic responses was not modulated by age or gender.

#### Associative strength

Considering all related responses in Experiments 1 and 2, 38 word pairs were represented in both experimental modalities as responses given by 2+ 3-year-olds for the same cue words. Ten of the word pairs, or 26%, are not represented in adult associative norms. The full list of word pairs found in all responses of both versions of the task can be found in Appendix Table 14.

For first responses, 13 word pairs were represented in both experiments (see Appendix Table 15). One of these word pairs was not represented in adult associative norms (7.69%).

The associative strength for related word pairs (in first responses) did not differ between Experiments 1 and 2, *t*(11) = 0.02, *p* = .98, 95% CI [−0.07, 0.07], with the average associative strength in the online version (*M*= 0.24, range = 0.17–0.42) equal to that in Experiment 1 (*M* = 0.24, range = 0.12–0.40). Word pairs are associated to an equal degree when the task is administered by a parent at home, or when done online.

### Discussion of Experiment 2

Experiment 2 clearly demonstrates that conducting a WA task online with 3-year-olds is a feasible and valid way to deliver this task, with evidence that it generates the same proportion and type of responses as when administered by a parent, in a home setting. There was no effect of age, which is likely because the age range is too narrow to observe a solid effect, as in Experiment 1.

Rate of response was comparable in Experiments 1 and 2, but also the type of response, with syntagmatic responses favoured in both versions of the task. Parental report of the WAs produced by their children was accurate 92% of the time, suggesting that it is an objective and reliable way to record the responses to a free association task in children, making it a comparable modality to the at-home version of the task.

In terms of the exact associated responses generated to the cue words by two or more children, we saw a replication of 38 word pairs from Experiment 1 (total = 432 pairs) and Experiment 2 (total = 72 pairs), when counting all responses given. For first responses only, 13 word pairs appeared in both experiments. There was no difference in the associative strength of these 13 word pairs when the experiment was done with a parent or when done online. The fact that so many word pairs were found in both experiments suggests that these might be particularly robust and thus more reliable for use in experiments investigating development of the lexical-semantic system. To investigate this claim, Experiment 3 will test these WAs in a priming experiment with a new sample of children.

## Experiment 3

To test the strength of association in the unique child WAs found in Experiments 1 and 2, Experiment 3 employs a receptive task. An online adaptation of the primed intermodal-preferential looking (IPL- see Arias-Trejo & Plunkett, 2009; Jardak & Byers-Heinlein, 2019; Styles & Plunkett, 2009) paradigm was developed for this purpose, after first validating an online word recognition IPL task (Nguyen, Fitzpatrick, & Floccia, 2024). Experiment 3 compared the magnitude of a semantic priming effect between child-specific associations, adult-specific associations, and associations found in both adults and children. Based on the findings in Experiments 1 and 2, it was hypothesised that adult WAs not represented in the child WA data may not show any semantic priming effect, or the effect may be smaller in magnitude compared to the word pairs found in children’s associations. In contrast, child-specific associations and those represented in both child and adult WA data were expected to show a consistent priming effect.

A stronger effect of priming in child-specific word pairs might indicate stronger receptive knowledge of these than productive knowledge (as measured in the WA task) or simply that a child’s attention will be maintained for longer for the unique child WAs since their experience of the world at the age of three is represented in these word pairings.

### Method

#### Power analysis and sample size calculations

A power analysis calculation was performed using an effect size extrapolated from Jardak and Byers-Heinlein (2019). The effect size showed that a sample size of 39 participants would be sufficient with 80% power^10^.

#### Participants

Forty 3-year-old healthy, English monolinguals were tested (19 girls, 21 boys). The average age of participants was 37 months 3 days (range = 35 months 3 days to 39 months 6 days). Productive vocabulary size was measured using the word list component of the MacArthur-Bates CDI III (Dale et al., 1998). The mean vocabulary score was 85/100. A further four participants were tested but excluded due to technical issues during testing.

#### Materials

Forty-eight common, highly imageable nouns were selected which are in the productive vocabularies of 3-year-olds (as demonstrated in Experiments 1 and 2). Nouns were selected either from the noun–noun WAs produced by 3-year-olds in Experiment 1 and/or Experiment 2 which had high FSG, or from the noun–noun WAs documented as having a high FSG in adults (Moss & Older, 1996; Nelson et al., 2004) and which have been selected for use in infant studies exploring semantic development (see Appendix Table 16 for the specific studies consulted). This resulted in three prime-target conditions: (i) unique child associations documented in the WAs of 3-year-olds (Experiments 1 and 2), (ii) validated adult associations (i.e. word pairs documented in both the adults’ WAs and the WAs of 3-year-olds), (iii) unvalidated adult associations (i.e. only found in the adult data, not in 3-year-olds’ associated responses). There were four trials per condition and 12 control/unrelated trials. Word pairs in unrelated trials had no attested associative or taxonomic relation, nor did distractor/target pairings in all trial types. Word pairs did not share phonological onset/rhyme. The full list of stimuli can be found in Appendix Table 16.

Twenty-four photographs of real objects were chosen to act as visual stimuli. Each visual stimulus was cut out of its background and presented centrally on a 50% grey background. The 24 images were seen twice by each participant: once as the target, and once as a distractor, appearing in different blocks to avoid an effect of repetition. The presentation side of the target was counterbalanced across participants. Each prime/ target word was individually recorded as auditory stimuli by a female speaker with a neutral British south-west accent, in a child-directed manner. Three neutral carrier phrases, i.e. ‘I want a/an…’, ‘I have a/an…’, ‘I saw a/an…’, were recorded in the same manner. The carrier phrase and prime word were concatenated into a single audio file for each trial. The target words were presented in isolation. Auditory and visual stimuli were presented using the experimental platform, Gorilla Experiment Builder. Four list orders were created to counterbalance presentation side of the target image. Block order was also counterbalanced. No 3-year-old saw more than two consecutive trials from the same relatedness condition.

#### Procedure

An information sheet about the study was emailed along with instructions for the study and a unique link to the Gorilla Experiment Builder website. A time was arranged for the parent to access the link when a researcher was available by email for questions or assistance.

The procedure replicated a previous asynchronous online experimental design (see Experiment 1, Nyugen et al., 2024: https://app.gorilla.sc/openmaterials/626885) in terms of pre-testing components, which included: eligibility checks, consent, collection of participant and demographic information, and instructions on how to position the child and how to run the experiment. The testing itself was procedurally different and is explained below.

Each trial began with a smiley fixation point in the centre of the screen for 1000 ms to focus the child’s attention to the middle of the screen. This was replaced by a blank screen and the carrier phrase embedded with a prime word (e.g. ‘I saw a… cat’) played auditorily. An inter-stimulus interval (ISI) of 200 ms was then followed by the target word (e.g. ‘dog’) and a stimulus onset asynchrony (SOA) of 400 ms (see Jardak & Byers-Heinlein, 2019) at which point two images appeared: one on the left-hand side of the screen, and one on the right. One of the images was a referent to the target word, and one was a distractor image. Both images remained on screen for a further 2600 ms. After 12 trials, a short animation was played to maintain the child’s interest. The second block of 12 trials then followed automatically. The experiment ended with a short animation. The parent could exit the task at any point by clicking on the ‘Exit’ button.

Parents completed a word checklist for the experimental words to test that the child was familiar with them, as well as completing the vocabulary component of the CDI III at the end of the procedure.

### Results

#### Data processing and analysis

Using university-developed bespoke software, webcam recordings of individual calibration and experimental trials were uploaded and automatically split into 50 ms frames. Calibration recordings were checked first to understand the looking behaviour of an individual (e.g. subtle/obvious saccades, the orientation of the screen in relation to the child’s position), and to validate that looks were being made to the side of target image presentation.

Each video of a trial was played in full, with audio, before analysis began. Since there was no recording of the visual stimuli in the video, hearing the audio did not influence manual coding of the eye gaze as the target location was unknown. This pre-analysis step served two purposes. First, it enabled us to check that the target word had been presented, with no significant delay in the Gorilla command to begin webcam recording. A second reason was to understand a participant’s looking pattern and head movement, to help when coding for left/right looks.

For experimental trials, the primary coder manually marked for each 50 ms frame if a child was looking left, right, on-screen but at an indeterminate location (which also accounts for saccades across the screen), or off-screen, using four keys on the keyboard. The coding was automatically saved in a .csv file which was later imported into R for analysis. A second coder coded a 10% subset of the data to test for rater reliability. Inter-rater reliability agreement between coders was 91% with a Cohen’s kappa κ of 0.80, indicating substantial agreement.

Trials were excluded if (i) a participant failed to look at the screen for a minimum time of 750 ms (or 15 frames, each measuring 50 ms) as per Jardak and Byers-Heinlein (2019) on each trial; (ii) the length of a given trial was under 2500 ms, as this signified that a technical error must have occurred; (iii) if a parent had marked either the prime word or target word as unknown to the child. Trials with webcam recordings without audio were excluded if the parent could not verify that sound had been played during the experiment. A participant was excluded if fewer than 50% of related and unrelated trials were available for analysis after excluding individual trials based on the above criteria. Analyses were completed in RStudio (1.4.1717 R Core Team, 2021), using R tidyverse (Wickham et al., 2019), and dplyr (Wickham et al., 2023) packages.

#### Descriptive statistics

Out of a possible 960 trials (a maximum of 24 trials for each of the 40 participants), a total of 920 trials were included for analysis. Reasons for exclusion were due to insufficient trial length (11 trials or 1% of trials); inattentiveness (<750 ms spent looking at the screen per trial: 11 trials or 1% of trials); prime or target word unknown to child (8 trials or 1% of trials); technical error (10 trials or 1% of all trials). No participants had to be replaced due to not meeting the minimum threshold number of trials, per condition.

The average number of valid trials per participant was 23 (*SD* = 1.99). This high number indicates children were very engaged in an online looking task when administered in the home. There was no effect of gender on response rate, *t*(38) = .96, *p* = .35, 95% CI [−0.67, 1.88]. Out of the four trial types, participants completed an average of 3.85/4 (*SD* = 0.59) trials for unique child word pairs, 3.8/4 (*SD* = 0.69) trials for validated adult word pairs, 3.75/4 (*SD* = 0.59) trials for unvalidated adult associations, and 11.65/12 (*SD* = 0.86) trials for unrelated word pairs.

#### Proportion of looking time to the target

The window of analysis was set at 200–2000 ms which coincides with visual stimulus onset, an allowance of 200 ms for an initial saccade, and a free-looking period of 1800 ms^11^. The proportion of looking time (PLT) towards the target visual stimulus, relative to the distractor stimulus, was calculated as the dependent variable for each trial as: PLT to target/(PLT to target+PLT to distractor).

**Fig. 4:**
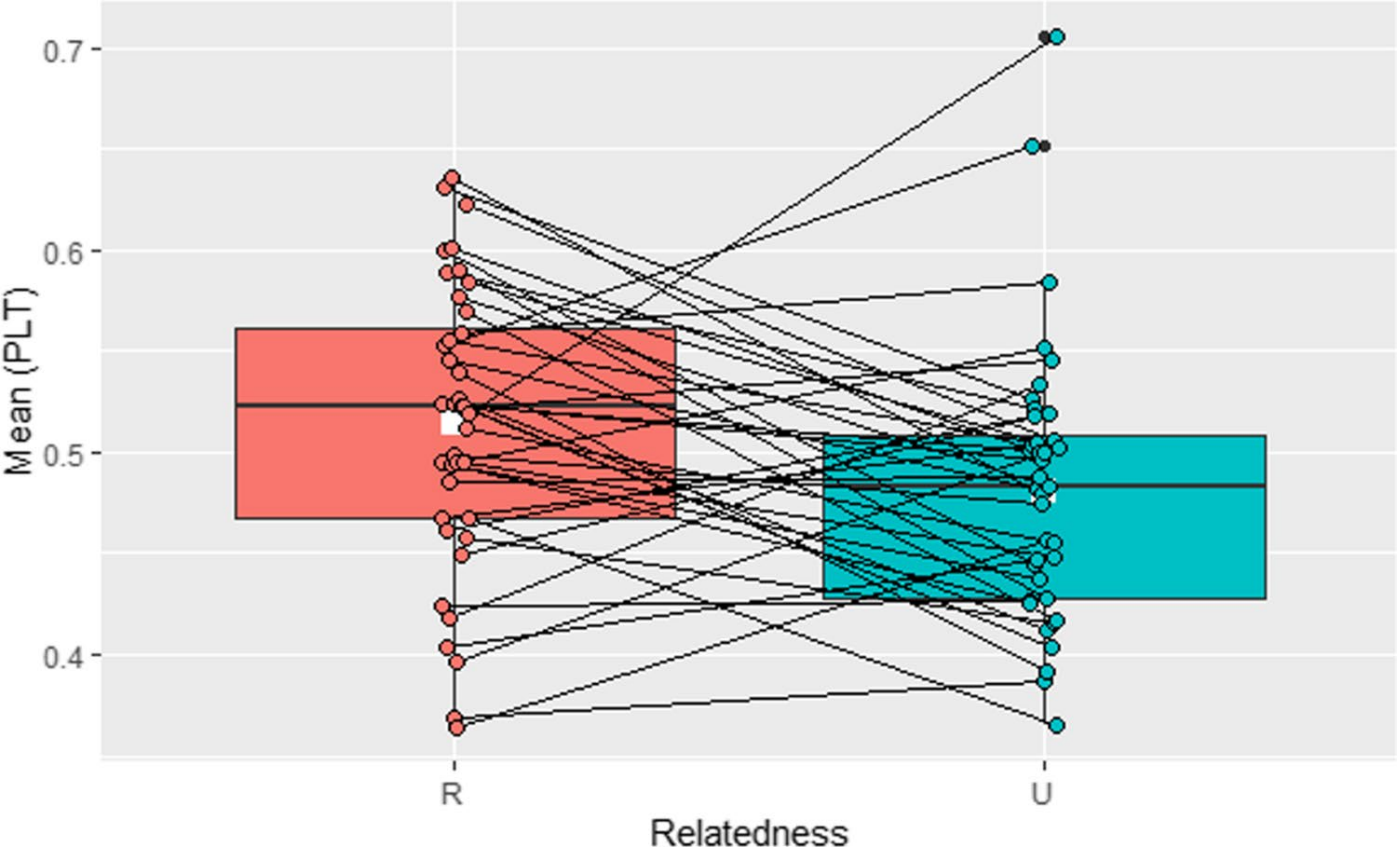
Experiment 3. Proportion of looking to a target visual stimulus on semantically related (red) and unrelated (blue) trials in an online semantic priming study on 3-year-olds (white square = mean in each condition)

A two-tailed, paired *t*-test was run on related and unrelated trials, showing that 3-year-olds looked significantly longer on related trials (*M* = 0.51, *SD* = 0.07) than on unrelated trials (*M* = 0.48, *SD* = 0.07), *t*(39) = 2.39, *p* = .02, *d* = .38, 95% CI [0.01, 0.06] (see Fig. 4).

A follow-up, one-sample *t*-test was performed to investigate whether looking was above chance (0.5) on related and unrelated trials. Comparisons to chance (0.5) with PLT indicated that 3-year-olds did not look significantly above chance in related, *t*(39) = 1.28, *p* = .10, 95% CI [0.50, Inf], or unrelated trials, *t*(39) = −1.76, *p* = .96, 95% CI [0.46, Inf].

In sum, the mean looking patterns of 3-year-olds indicated some sensitivity to the different relationship between words, demonstrated by a target preference when trials were related. However, there was no evidence of target recognition which is usually indexed by above-chance looking. The target not being recognised in unrelated trials replicated previous lab-based studies (e.g. Arias-Trejo & Plunkett, 2009; Styles & Plunkett, 2009), but the lack of target recognition on related trials was unexpected.

#### Association type

To examine the effect of association type (unique child, unique adult, adult and child, and unrelated), a one-way, repeated measures ANOVA was run on PLT with association type as a fixed factor. The PLT was statistically different for association type, *F*(2.57, 100.1) = 13.13, *p* <. 0001, generalized η^2^ = .19.

**Fig. 5:**
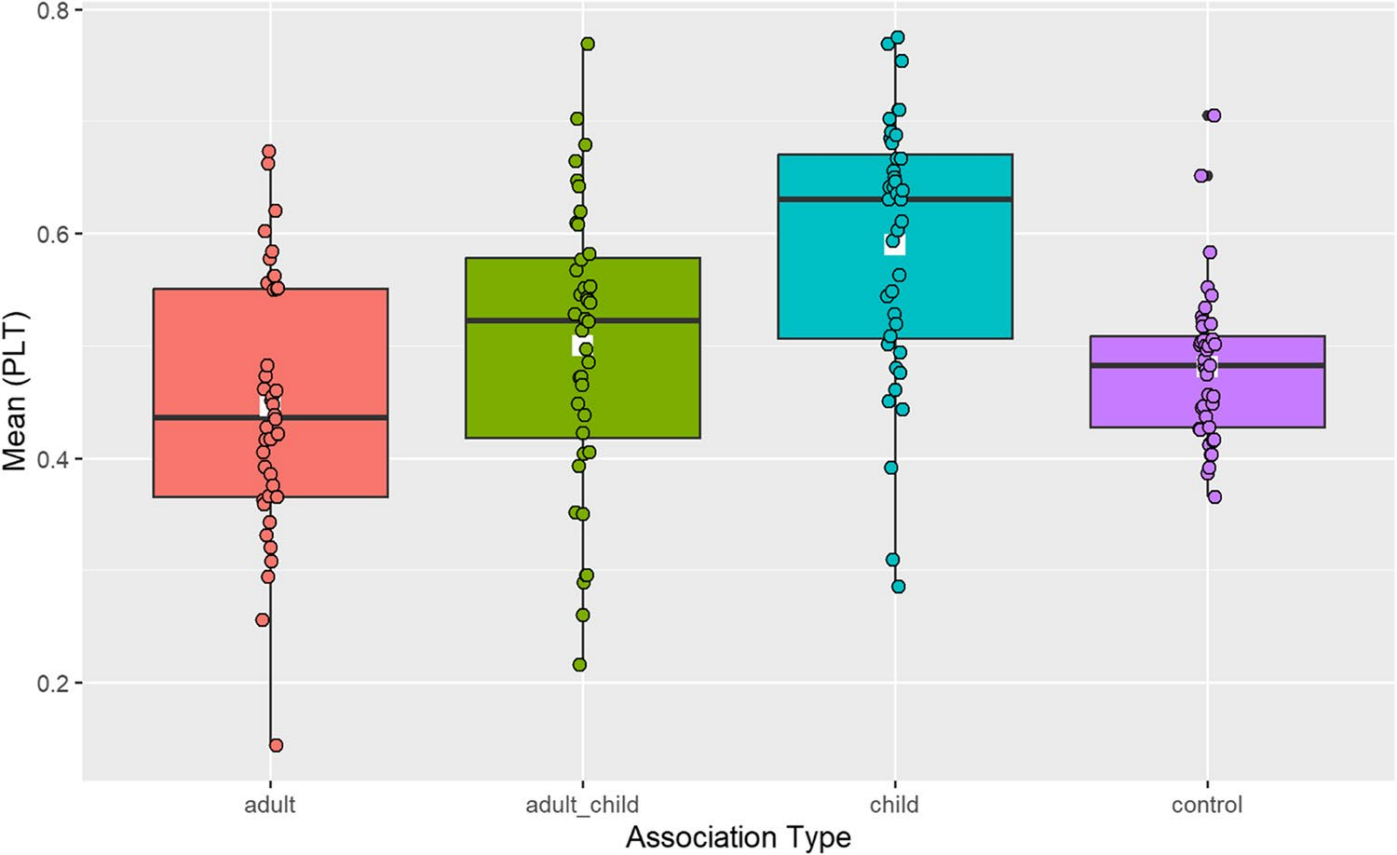
Experiment 3. Proportion of looking time to the target by word association type in 36–39-month-olds doing an online semantic priming task (white square = mean of each condition)

Planned pairwise comparisons were performed with a Bonferroni adjustment to identify the locus of the difference. Post hoc analyses revealed that the PLT to the target for child-specific associations (*M* = 0.59, *SD* = 0.12) differed significantly to adult-specific associations (*M* = 0.45, *SD* = 0.12; *p* < 0.0001), 95% CI [−0.21, −0.08]; to adult-child associations (*M* = 0.50, *SD* = 0.13; *p* = .003), 95% CI [−0.16, −0.02]; and to control trials (*M* = 0.48, *SD* = 0.07; *p* < .0001), 95% CI [0.04, 0.17]. Other pairwise comparisons were not statistically significant. These data are visualised in Fig. 5.

Comparisons to chance (0.5) with PLT indicated that 3-year-olds looked significantly above chance in trials with child-specific associations (*t*(39) = 4.82, *p* < .0001), but not in trials with adult–child associations (*t*(39) = −0.01, *p* = .5), adult-specific associations (*t*(39) = −2.96, *p* = .1), or unrelated trials (*t*(39) = −1.76, *p* = .96). Together this shows that children looked longer at the target when the prime-target word pair had been generated in the WA task (see Experiments 1 and 2), compared to other WA types tested here. The lack of above-chance looking for adult or adult–child associations and unrelated word pairs suggests that no target recognition was indexed.

A correlation between CDI scores and priming difference scores, which were calculated by subtracting the PLT on unrelated trials from the PLT on related trials per child, as per Jardak and Byers-Heinlein (2019), showed no relation between productive vocabulary size and priming, *r*(37) = .06, *p* = .7.

#### Paradigmatic/syntagmatic analysis

**Fig. 6:**
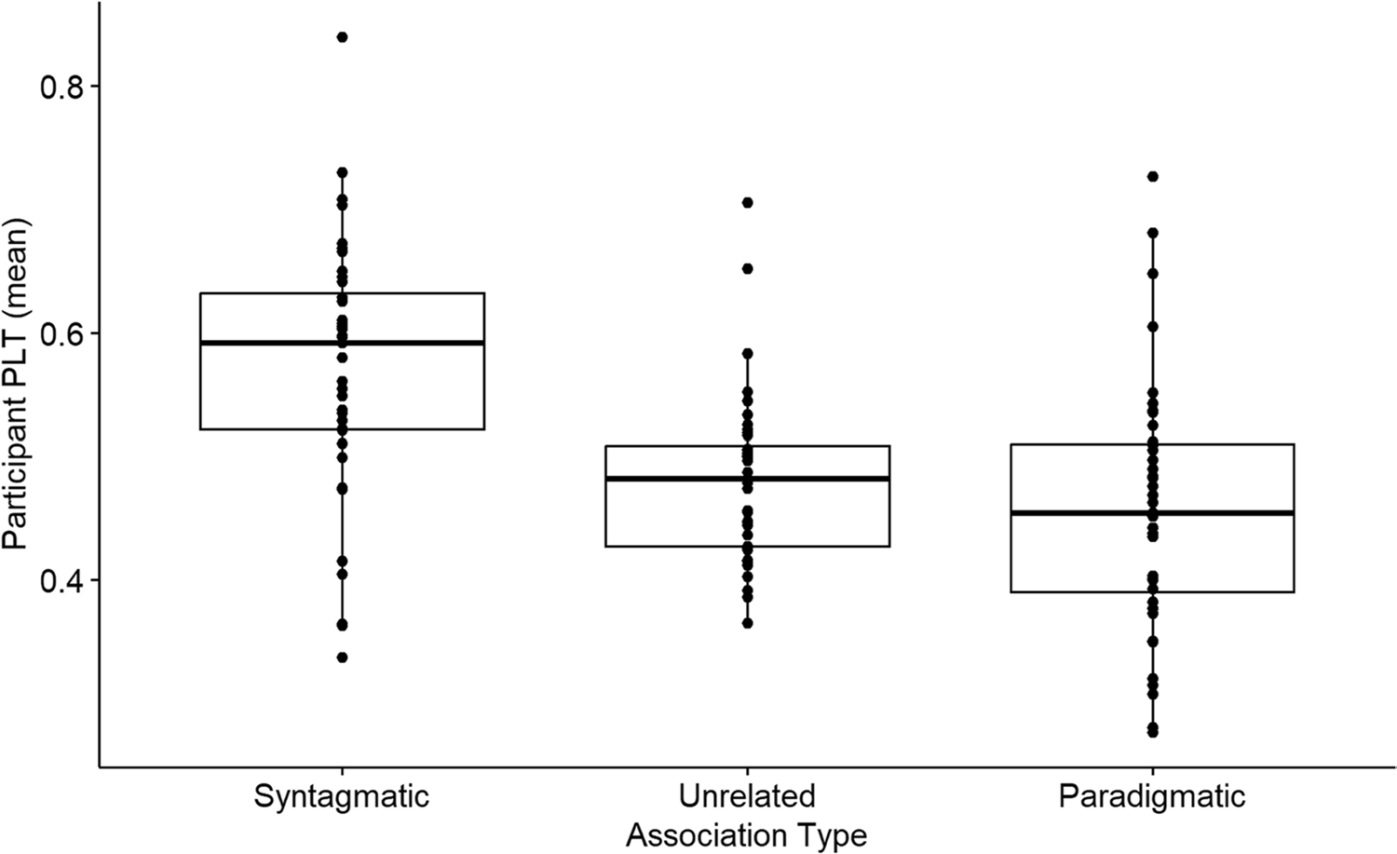
Experiment 3. Proportion of looking time to the target by paradigmatic, syntagmatic, or unrelated association type in 36–39-month-olds doing an online semantic priming task

We re-coded related word pairs as paradigmatic/both or syntagmatic (according to the definitions used in Experiment 1—see the Coding for Response Type section), rather than using our original unique child, unique adult, adult and child, related response types. Re-coding was done from a child’s perspective (i.e. whether the association is documented in the responses to Experiments 1 and 2 in this paper) rather than from an adult’s perspective and based on adult norms. For example, *boots*–*puddle* was coded as syntagmatic, whereas looking at adult norms to guide coding, this would not have appeared as associatively related. The mean PLT per paradigmatic/syntagmatic association type is visualised in Fig. 6.

To examine the effect of paradigmatic/syntagmatic association type, a one-way, repeated-measures ANOVA was run on PLT with paradigmatic/syntagmatic association type as a fixed factor. The PLT was statistically different for paradigmatic/syntagmatic association type,

*F*(1.72, 67.16) = 18.03, *p* < . 0001 , generalized η^2^ = .23.

Planned pairwise comparisons were performed to identify the locus of the difference. Post hoc analyses revealed that the PLT to the target for syntagmatic associations (*M* = 0.57, *SD* = 0.10) differed significantly to paradigmatic associations (*M* = 0.46, *SD* = 0.10; *p* < .0001), 95% CI [−0.17, −0.07], and to unrelated word pairs (*M* = 0.48, *SD* = 0.06; *p* < .0001), 95% CI [0.04, 0.14]. A pairwise comparison of paradigmatic and unrelated trials was not significantly different (*p* = .60), 95% CI [−0.08, 0.03].

#### Time-course analysis

**Fig. 7:**
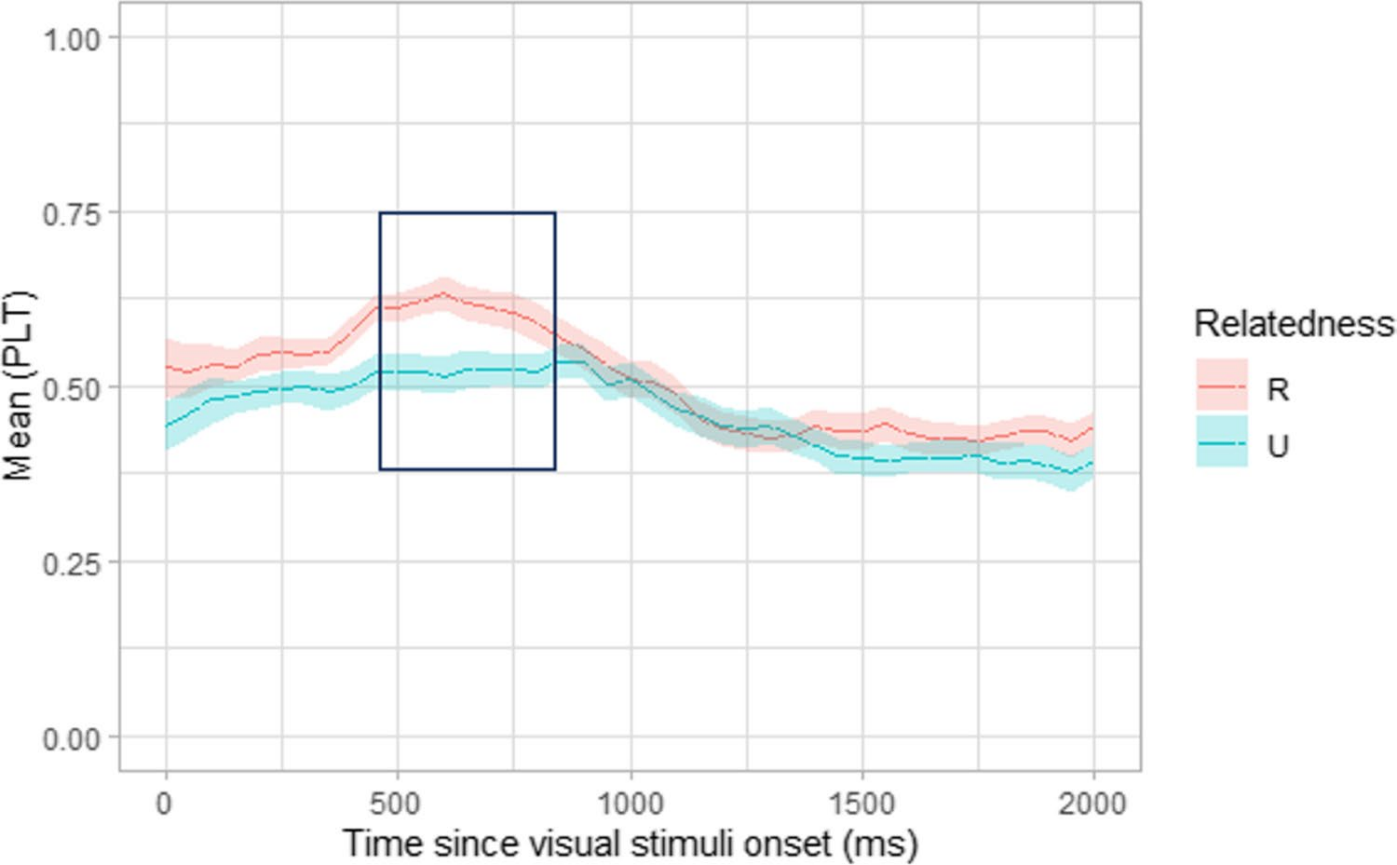
Experiment 3. Time-course of looking behaviour in 36–39-month-olds for semantically related and unrelated trials with the significant divergence in behaviour indicated by a boxed area

Looking behaviour over time was interrogated using a time-course analysis to understand where 3-year-olds looked throughout the 1800 ms looking period. The PLT to the target for related and unrelated trials was averaged across participants for each 50 ms time bin and plotted using the R package eyetrackingR (Forbes et al., 2021; see Fig. 7). Visual inspection suggests that the curves start to diverge at approximately 125 ms.

To determine where any difference in looking behaviour occurred on related and unrelated trials during the time-course of word recognition, a non-parametric statistical cluster analysis was performed (see Maris & Oostenveld, 2007), which has been successfully employed by various studies investigating preferential looking (Floccia et al., 2020; Von Holzen et al., 2019; Von Holzen & Mani, 2012). Paired *t* tests were run for each time bin, followed by identifying clusters with significant *t* vales and comparing these to a Monte Carlo distribution. Comparisons using the time-course analysis revealed a significant difference in looking behaviour between 450 and 850 ms post visual stimulus onset (cluster *t* statistics = 27.99, Monte Carlo *p* < .001) between related and unrelated trials, with the unrelated condition showing reduced looking in this period compared to related trials. This area is marked by a box in Fig. 7. This analysis suggests that the priming effect, as indexed by the difference in PLT in the related and unrelated conditions, occurs at around 450 ms after target onset.

### Discussion of Experiment 3

The main aim of Experiment 3 was to ascertain whether the unique child WAs found in Experiments 1 and 2 would demonstrate a measurable difference in a receptive semantic priming task. To explore this, we compared PLT for each WA type: unique child, unique adult, child and adult, and unrelated. The results clearly demonstrated that the priming effect was modulated by WA type. Related word pairs with the highest PLT were those taken from the productive vocabularies of 3-year-olds, tested in a WA task (Experiments 1 and 2). This WA type was the only of the four types tested with an above-chance probability of looks towards the target image. The finding that PLT for child-specific WAs differed significantly to the two other WA types (adult-specific, child and adult) suggests that an effect of semantic priming only occurred in the combined related data due to the associative boost provided by the child-specific WAs. However, the absence of above-chance looking when all three related WA types were combined might suggest that an online modality is not sensitive enough to capture general priming effects, particularly for WAs not robust in a child’s lexical-semantic system (i.e. some of those stemming from adult associative norms).

After performing a time course analysis on looking behaviour, we found a significant difference between PLT on related and unrelated trials. This indicates that children spent longer looking at the target image on related trials. Visual inspection reveals that looking times to the target raise to above 60% in the related trials, while they remain at 50% for the unrelated ones. We observed that children made saccades to the target stimulus before 200 ms. On average, first looks were slightly above chance for related trials but not for unrelated trials. We found a significant finding between 450 and 850 ms, where 3-year-olds looked above chance at the target more on related trials, than unrelated trials. Thus, while an effect of above-chance looking was absent when analysing the averaged PLT per trial type, the pattern of findings from this time course analysis suggests that children did recognize the target on related trials.

As hypothesised, WAs not found in the productive vocabularies of 3-year-olds, but prominent in the associated responses of adults performing a WA task, did not show a strong effect of priming in this experiment. This deserves attention, as many studies exploring the primacy of connections in the lexical-semantic system of infants have relied on associative norms from the adult literature to drive decisions regarding experimental stimuli for their studies. Studies which might not have seen a priming effect could be a result of stimuli selected, with the assumption that a WA in the adult lexical system is equivalently robust in the infant system. In experiments that did find a priming effect, further analysis on the stimuli selected could help inform other researchers on the best word pairs to select for infant studies.

A finding that we did not expect to see was the lack of a priming effect in child–adult associations, that is, word pairs documented in our own findings of Experiments 1 and 2 (for 3-year-olds) and in adult associative norms (Moss & Older, 1996; Nelson et al., 2004). One explanation for no semantic priming in child and adult WAs may be the syntagmatic nature of the child-specific WAs compared to the more paradigmatic child and adult WAs. The most reliable effect of semantic priming has been found in words both taxonomically and associatively related (e.g. *chair*–*table*) due to the associative relatedness providing a ‘priming boost’ (infants: Arias-Trejo & Plunkett, 2009; adults: McRae & Boisvert, 1998; Perea & Rosa, 2002). While evidence exists to show that pure taxonomic relationships can evidence a priming effect in young children (Arias-Trejo & Plunkett, 2013), this was in an in-lab testing context, while our Experiment 3 was online.

We interrogated the potential syntagmatic/paradigmatic explanation by re-coding prime-target pairs as paradigmatic or syntagmatic and re-analysing the data. We found a significant difference between the PLT for syntagmatically associated word pairs compared to paradigmatic or unrelated pairs. This presents a confound between child-specific WAs and a syntagmatic advantage. It could be that the child-specific WAs showed better priming because they are syntagmatic, but the fact that they have the strongest FSG in the data is also certainly because they are syntagmatic. This confound can potentially never be solved since most child-specific associations are syntagmatic.

Taken together, Experiment 3 replicates in-lab findings in as far as a semantic priming effect was measured, but the lack of above-chance looking on (combined) related trials requires further investigation to determine whether the finding was unique to this experiment, or whether it more broadly represents an issue with the sensitivity of an online priming procedure.

## General discussion

In three experiments, we tested the strength and types of word–word relationships in English-speaking children as young as 3 years old. Experiment 1 used a WA methodology administered by the parent in the home setting, and Experiment 2 replicated the method in an online format. Responses given by 3-year-olds were compared to the responses found in adult associative norms. Experiment 3 tested how the WA responses given by children, adults, or both groups indexed a semantic priming effect to determine whether some word–word relationships are more consolidated in a 3-year-old’s lexical semantic system.

Conducting a WA task with 3-year-olds at home and online generated the same proportion and type of responses for a subset of stimulus words. This is in line with our hypothesis and indicates that the parent administering the task did not confound the findings. In fact, our attempt to increase the engagement of the task by using puppets to demonstrate the task rather than a parent did not result in improved performance either. We think that this might be due to the parent’s continued involvement even when the task was done online. The parent dictated the pace of the task, was responsible for recording the child’s responses, and was also instructed to encourage second and third attempts at the task for each of the 10 cue words. Thus, the parent was an intrinsic part of the process in both modalities and perhaps was the key contributor to engagement levels and supporting associated response types.

The fact that many WAs in children^12^ were not found in adult norms might be indicative of the transitory nature of the immature lexical-semantic system. Some adult associations might not form in infancy; instead, these findings suggest that there are unique WAs at 3 years of age which may be replaced by other, more adult-like associations, with increased age and life experience. This could occur in parallel to a subset of word pairs, shown to exist in both children and adults, though the strength of these associations differs.

For example, in a semantic priming study on children, Arias-Trejo & Plunkett (2009) demonstrated that associative relatedness can provide a ‘priming boost’ for word pairs which are taxonomically related. The authors defined associative word pairs as those taken from adult word association norms (Kiss, 1975; Moss & Older, 1996) without categorical relatedness. Taxonomically related word pairs were defined as objects with the same superordinate term (e.g. clothes, sock–pants) without associative relatedness. Thus, when considering the primacy of word–word relationships in the emerging lexical-semantic system, associative links might support the structuring of more complex, taxonomic connections and explain why they are more prevalent in the associated responses of 3-year-olds. Associative links that exist in memory may arise due to a child’s early experience of a conjunction of events: experience of the real world (e.g. playing with toys in the bath) and their exposure to recurring words that are uttered during those moments. Therefore, the links between toys and bath, for example, might be of two kinds: links between visual representation and lexical forms. In contrast, taxonomic links may emerge from a re-representation of meaning within an existing lexicon, based solely on abstract knowledge. This might suggest why some WA studies on children note a ‘syntagmatic-paradigmatic’ shift (White, 1985), evidencing a change in children’s responses to a WA task as they age. Our findings clearly indicate that WAs produced by 3-year-olds were more syntagmatic in nature, and when these were tested in a priming task, the word pairs with a syntagmatic relationship indexed a larger priming effect than words with a paradigmatic relationship.

According to Fitzpatrick et al. (2013), referencing WAs that have not been taken from the target population might not acknowledge the unique characteristics of the population of interest. This might be true of the WAs found in the children of this study and missing from the adult literature. Therefore, one must be cautious when interpreting the WAs found in adult norms, as the absence of a WA in adult associative norms is not necessarily a reliable indicator of its absence in the developing lexical-semantic system.

The absence of some of the strongest child WAs in the associative norms of adults is of relevance to the wider research field. Studies designed to investigate semantic development in infants rely on the WAs documented in adult norms when selecting appropriate stimuli (i.e. prime and target word pairs). For example, the word pair ‘teddy–bed’ from the WAs found in 3-year-olds is not present in adult norms. This word pair intuitively constitutes a strong association in the mind of a child, though relying on adult norms would not capture it as a suitable pair for use in an experiment. This example serves to highlight the importance of considering the most child-appropriate word pairs for use in experiments investigating the emergence of semantic meaning in infancy.

## Limitations

One limitation of this research is the fact that we did not do a direct comparison of syntagmatic adult associations and syntagmatic child associations. This is something we hope to explore in future work. Due to the difficulty in directly comparing syntagmatic and paradigmatic WAs in children, because the children in this study did not produce many of the latter, we might look to the adult data or studies on older children to explore this further.

## Conclusion

The sample of 3-year-olds tested in this study clearly share some of the WAs found in adult associative norms, but have their own, more child-specific associations, which can be stronger than word pairs in the adult literature. These child-specific word pairs are predominantly syntagmatic, and they index a larger semantic priming effect compared to paradigmatic word pairs.

This suggests a more reliable source of WAs for use in semantic priming studies needs to come from the WAs documented in children rather than adults, and ideally in children as close in age to the population being tested. The Appendices attached to this paper provide a resource of associatively related word pairs which reflect the associated responses to cue words produced by two or more 3-year-olds engaged in a free association task. Many of these word pairs comprise imageable noun–noun combinations which can be consulted for stimuli selection when designing studies investigating semantic development in young children. These word pairs reflect language production, and since production succeeds language comprehension, which is what studies investigating semantic development typically test, it is the closest we might get to knowing the precise WAs children form as their lexical-semantic system undergoes development.

## Appendices

**Fig. 8:**
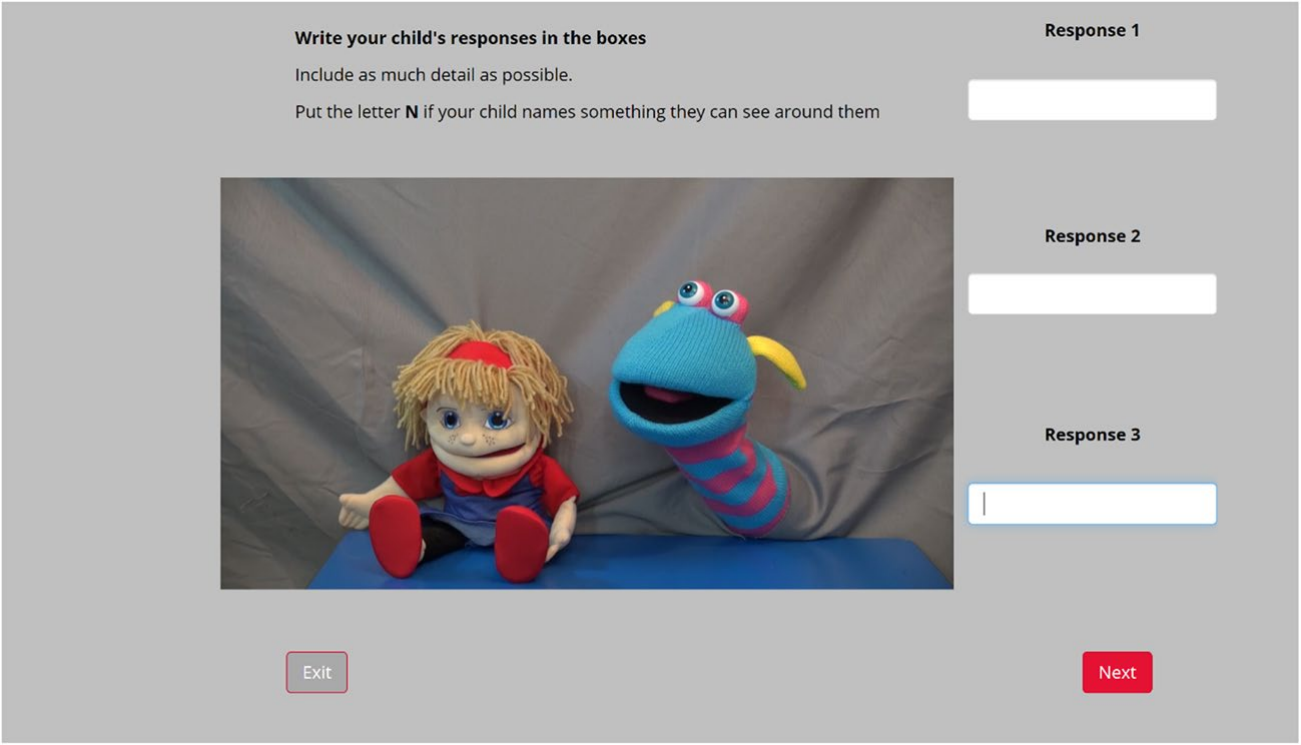
Experiment 2. Screenshot of the online WA task

See Tables 3, 4, 5, 6, 7, 8, 9, 10, 11, 12, 13, 14, 15, 16 and Fig. 8

**Table 3:** Experiment 1. The percentage of 18-month-olds knowing the words used as cues in the word association task

Word	OCDI % 18mths	UKCDI % 18mths
aeroplane/plane	81	72
apple	75	82
arm	56	75
ball	98	99
balloon	84	83
banana	91	94
bath/bathtub	94	98
bed	85	97
bee	60	69
bib	75	66
bicycle/bike	69	72
bin	70	83
bird	88	88
biscuit	88	86
boat	62	69
book	95	98
boots	54	65
bottle	65	80
bowl	58	77
box	48	63
bread	72	77
brush	72	77
bubbles	61	85
rabbit	77	77
bus	69	81
butterfly	54	63
cake	54	74
car	95	97
carrots	48	74
cat	94	94
cereal	26	67
chair	80	95
cheese	63	78
chicken	58	72
coat	77	90
cot	70	68
cow	83	82
cup	79	83
dog	98	99
doll	60	73
door	87	96
duck	90	86
ear	84	83
elephant	54	70
eye	86	96
finger	82	79
fish	75	81
flower	77	68
feet	70	92
fork	46	65
frog	56	68
garden	73	72
hair	91	86
hand	77	85
hat	87	89
head	75	89
high chair	68	78
horse	76	78
house	57	78
key	74	81
leg	59	81
lion	65	79
lorry/truck	61	58
monkey	57	90
mouse	54	67
mouth	76	91
nappy	92	98
nose	94	94
orange	37	63
park	38	72
pasta	35	60
peas	47	70
pen	53	70
pig	77	82
plate	52	66
pushchair/buggy/stroller	77	82
pyjamas/Pjs/jim jams	54	80
settee/sofa/couch	48	74
sheep	69	76
shoes	99	97
slide	59	72
sock	92	91
spoon	77	76
stairs	81	86
swing	64	68
table	64	78
teddy/teddy bear	85	91
phone/telephone/mobile	87	91
tiger	50	72
toast	70	84
toe	71	76
tooth/teeth	75	85
toothbrush	86	94
towel	57	68
toy	60	82
train	66	81
tree	69	78
trousers/pants/britches	55	76
television/telly/TV	77	89
window	63	78

**Table 4:** Experiment 1. Word association task instructions and script for parents

3. Complete the test using the script below Follow the script as closely as you can. Say all 3 examples. For every word in the list, try to get up to 3 different responses. 1 response per word is absolutely fine though. Try do all 10 words in one go if possible. There are no right or wrong answers! Have fun!
Script “We’re going to play a game to see how quickly you can say a word that is connected to a word that I say. If I say KITCHEN you might say BREAKFAST. (Example 1) If I say MUMMY you might say DADDY. (Example 2) If I say DRINK you might say WATER. (Example 3) Okay, are you ready? What do you think of if I say …? (Response 1) And another word? (Response 2) And another?” (Response 3)

**Table 5:** Experiment 1. Categories for coding participant responses

Category	Description
0	No response given/ “I don’t know”/ “I don’t want to play”
1	Recognised association (i.e. what an adult might say in response to the word)
2	Association unique to individual (based on parental comments- given in brackets if there are any; or when referencing own life e.g. “my car”)
3	Association arising from a previous response given (e.g. PIG- 1^st^= mud, 2^nd^= straw, 3^rd^= moss. The 2^nd^ and 3^rd^ responses relate to the 1^st^ response ‘mud’ rather than the cue word PIG)
4	Related in a general/wider sense (i.e. not an obvious association but a logical connection e.g. trousers- people)
5	Repetition of the cue word/ repetition of a response already given
6	Naming something in the immediate environment (this will be noted in brackets)
7	An unclear association (i.e. cannot be coded 1–6 or 8–10)
8	Rhyme (e.g. CAR - bar)
9	Sounding out (e.g. APPLE – ‘a’ for apple)
10	Action/mime or sound to indicate cue word (e.g. LION–roar)

**Table 6:** Experiment 1. All related responses (first, second, and third attempts) produced by 2+ children in the parentally administered WA task

	Cue	Response	No. participants receiving cue (G)	No. participants producing response (P)	Associative strength: FSG (P/G)	Idiosyncratic responses
1	apple	Eat/ eat it/ you can eat it	38	7	0.18	19
		Juice in it/ apple juice	38	2	0.05	19
		Pear	38	2	0.05	19
		Red	38	2	0.05	19
		yummy/ they are yummy	38	2	0.05	19
2	arm	Leg/ legs	43	4	0.09	19
		finger	43	3	0.07	19
		hand/ hands/ DEF: It's something that you make your hand grab something. Hand	43	3	0.07	19
		Body	43	2	0.05	19
		Elbow	43	2	0.05	19
		Head	43	2	0.05	19
3	ball	kick/ kick kick/ kicking	30	8	0.27	13
		football	30	4	0.13	13
		Throw/ throwing/ throw up high	30	3	0.10	13
4	balloon	pop	33	4	0.12	20
		holding/ Holding a balloon/ We hold them	33	3	0.09	20
		Party	33	2	0.06	20
		red	33	2	0.06	20
5	banana	Eat/ eat it/ eat the banana/ eating	32	5	0.16	21
		apple	32	2	0.06	21
		Yellow	32	2	0.06	21
		Fruit	32	2	0.06	21
6	bath	toy/ toys/ bathy toys/ put the toys in	40	5	0.13	22
		water	40	4	0.10	22
		to wash ourselves/ wash/ wash hair	40	3	0.08	22
		bubbles	40	2	0.05	22
		duck/ duckies	40	2	0.05	22
		splashing/ splash	40	2	0.05	22
7	bed	sleep/ to sleep	39	6	0.15	17
		teddy/ teddy bear/ Lambie (Teddy)/ cuddle up with teddies	39	5	0.13	17
		blanket	39	3	0.08	17
		cushion	39	2	0.05	17
8	bee	honey	35	5	0.14	16
		bumblebee/ Bumble Bee	35	3	0.09	16
		Flower/ flowers	35	3	0.09	16
		Fly	35	2	0.06	16
9	bib	Baby/ A Baby	36	6	0.17	27
		Food	36	2	0.06	27
		No bib/ No (She doesn’t wear a bib anymore, her decision. This ‘No’ is her saying no to wearing a bib.)	36	2	0.06	27
10	bicycle/ bike	bell	36	4	0.11	24
		ride/ riding	36	2	0.06	24
		scooter	36	2	0.06	24
		wheels	36	2	0.06	24
11	bin	Rubbish/ Rubbish in the bin/ put rubbish in it	35	7	0.20	13
		Smelly Bin/ smelly	35	2	0.06	13
		Lid	35	2	0.06	13
12	bird	fly	38	3	0.08	23
		Nest	38	3	0.08	23
		outside/ Bird outside	38	2	0.05	23
		feather/ feathers	38	2	0.05	23
13	biscuit	chocolate	33	4	0.12	15
		eat/ Eat! (shouts excitedly)/ eating	33	3	0.09	15
		Kitchen	33	2	0.06	15
		yummy	33	2	0.06	15
14	boat	Water/ In the water/ We was on a boat on water	35	5	0.14	16
		Sail/ sailing	35	3	0.09	16
		sea	35	3	0.09	16
15	book	Bedtime	32	4	0.13	12
		Read/ reading	32	4	0.13	12
		Story/ read story	32	4	0.13	12
		pictures	32	3	0.09	12
		pages	32	2	0.06	12
16	boots	Puddle/ puddles/ muddy puddles/ Splashing in muddy puddles	32	5	0.16	19
		Walk/ walking	32	3	0.09	19
		Feet	32	2	0.06	19
17	bottle	Water/ Water bottle	32	6	0.19	10
		Milk	32	4	0.13	10
		Cup	32	2	0.06	10
		Juice	32	2	0.06	10
		drink	32	2	0.06	10
		lid	32	2	0.06	10
18	bowl	breakfast	42	4	0.10	20
		food/ Tasty food	42	3	0.07	20
		dinner	42	2	0.05	20
		Shredded Wheat/ Shreddies	42	2	0.05	20
19	box	toys/ toys in it (obsessing over toys that morning!)	37	4	0.11	23
		Make (makes models from boxes)/ making/ make something	37	3	0.08	23
		Stuff/ Put stuff in it	37	2	0.05	23
20	bread	eat/ I eat it	39	3	0.08	23
		Toast	39	3	0.08	23
		butter	39	2	0.05	23
		honey	39	2	0.05	23
		kitchen	39	2	0.05	23
		Egg/ eggy	39	2	0.05	23
21	brush	hair/ Sophie’s long hair/ brush everyone hair/ Brush hair	32	6	0.19	19
		Teeth/ brush your teeth	32	5	0.16	19
		floor	32	2	0.06	19
22	bubbles	Pop/ DEF: They're something that pop. Pop the bubbles	46	8	0.17	19
		blow/ blowing/ Blow bubbles	46	4	0.09	19
		bath	46	3	0.07	19
		float/ float in the sky	46	2	0.04	19
		Water	46	2	0.04	19
23	bus	car/ cars	37	3	0.08	25
		train	37	2	0.05	25
		wheel/ wheels	37	2	0.05	25
		big/ big bus	37	2	0.05	25
		red	37	2	0.05	25
24	butterfly	wings/ yellow wings/ yellow and blue wings	30	4	0.13	19
		flying/ fly away	30	3	0.10	19
25	cake	birthday	35	4	0.11	20
		eat/ eat it	35	3	0.09	20
		chocolate/ choc	35	2	0.06	20
		sprinkles	35	2	0.06	20
26	car	Drive/ drive somewhere/ driving	36	3	0.08	18
		wheels/ Wheels to bump	36	3	0.08	18
		Beep/ beep beep	36	2	0.06	18
		Seat/ car seat	36	2	0.06	18
27	carrots	eat/ eat them/ eating	34	5	0.15	18
		Crunch crunch/ crunchy	34	2	0.06	18
		rabbit	34	2	0.06	18
28	cat	Dog	49	8	0.16	19
		Cat food/ food	49	2	0.04	19
		Elephant	49	2	0.04	19
		Meow	49	2	0.04	19
29	cereal	Milk/ Blue milk	39	5	0.13	23
		bowl	39	4	0.10	23
		eat/ eat cereal	39	3	0.08	23
		spoon	39	2	0.05	23
		Weetabix	39	2	0.05	23
		breakfast	39	2	0.05	23
30	chair	sit/ sit down	38	4	0.11	17
		breakfast	38	3	0.08	17
		eat/ you can eat	38	3	0.08	17
		Table	38	2	0.05	17
31	cheese	Eat/ eating/ eat it/ DEF: Easy- it's something that you eat and it's so squeezey. Apple	35	7	0.20	19
		Doggy/ dogs/ doggie	35	3	0.09	19
32	chicken	eat/ eating/ we eat it	38	4	0.11	27
		egg/ eggs	38	4	0.11	27
		Cock a doodle doo	38	2	0.05	27
33	coat	rain/ When it's just raining got to put your coat on	32	4	0.13	18
		red	32	3	0.09	18
		out/ going out	32	2	0.06	18
		cold	32	2	0.06	18
		hood/ hood on	32	2	0.06	18
		jacket	32	2	0.06	18
		Sleeve/ sleeves	32	2	0.06	18
34	cot	baby/ babies	28	7	0.25	14
		Sleep/ go to sleep	28	2	0.07	14
35	cow	moo	36	6	0.17	14
		milk/ ae some milk	36	5	0.14	14
		Pig	36	3	0.08	14
		farm	36	2	0.06	14
		fields/ In the field	36	2	0.06	14
		Goats (loves Pennywell)/ Daddy goat	36	2	0.06	14
36	cup	drink/ drink it/ Daddy drinking	34	8	0.24	7
		milk	34	4	0.12	7
		Water	34	4	0.12	7
37	dog	woof/ woof woof	39	6	0.15	22
		cat/ Kitty cat	39	5	0.13	22
		walk	39	2	0.05	22
38	doll	Chair	37	2	0.05	26
39	door	Open/ open it	34	5	0.15	21
		Shut	34	2	0.06	21
40	duck	Quack/ quack quack/ they go quack quack	38	11	0.29	18
		water	38	3	0.08	18
		swim/ swimming	38	3	0.08	18
41	ear	Earrings	33	3	0.09	17
		Listen/ listening ears	33	3	0.09	17
		Mummy/ on mummy	33	3	0.09	17
		eye/ eyes	33	3	0.09	17
42	elephant	Big	38	4	0.11	13
		trunk	38	4	0.11	13
		Ears	38	3	0.08	13
		Stomp stomp/ stomp	38	2	0.05	13
43	eye	I spy/ spy	37	4	0.11	20
		Nose	37	3	0.08	20
		eye lash/ eyelash	37	2	0.05	20
		ball/ balls	37	2	0.05	20
		head/ On my head	37	2	0.05	20
44	foot/ feet	toes	38	4	0.11	23
		shoes	38	2	0.05	23
		sock/ socks	38	2	0.05	23
		hands	38	2	0.05	23
		walk	38	2	0.05	23
45	finger	Hand/ hands	37	7	0.19	17
		nail/ nails	37	2	0.05	17
		Point/ pointing	37	2	0.05	17
		thumb	37	2	0.05	17
		Touch/ touch nose	37	2	0.05	17
46	fish	water/ lives in water	36	5	0.14	22
		eat/ eating	36	3	0.08	22
		fish finger/ fingers/ Eat fish fingers	36	3	0.08	22
		tank/ In the tank	36	3	0.08	22
		Swim/ swimming	36	3	0.08	22
		Sharks	36	2	0.06	22
47	flower	bee/ bees/ buzzy bee	36	4	0.11	18
		Grow	36	2	0.06	18
		petals	36	2	0.06	18
		Water	36	2	0.06	18
		daisy	36	2	0.06	18
		Pretty	36	2	0.06	18
48	fork	Knife	36	7	0.19	13
		Spoon	36	6	0.17	13
		Eat/ to eat	36	4	0.11	13
49	frog	ribbit/ Frog says ribbit	35	4	0.11	23
		Water	35	3	0.09	23
		Green	35	2	0.06	23
		jump/ Jump (and she jumps)/ jumps in	35	2	0.06	23
50	garden	trees/ apple tree	41	3	0.07	30
		Chair	41	2	0.05	30
		grass	41	2	0.05	30
		Pea	41	2	0.05	30
		trampoline/ Trampoline (has one in the garden)	41	2	0.05	30
		bee/ Bees in the garden	41	2	0.05	30
		Play	41	2	0.05	30
51	hair	brush	35	4	0.11	22
		Head	35	3	0.09	22
52	hand	fingers/ fingers	34	5	0.15	13
		foot	34	2	0.06	13
		Hair	34	2	0.06	13
53	hat	head/ It goes on your head	33	3	0.09	21
		Wear a hat/ wear it/ we can wear a hat	33	3	0.09	21
54	head	Hair/ Hair (pointing to his hair)/ hair on	36	8	0.22	19
		Ears	36	2	0.06	19
		Eyes	36	2	0.06	19
		Mummy head/ mummy	36	2	0.06	19
		brain	36	2	0.06	19
55	high chair	breakfast	35	2	0.06	18
		eat	35	2	0.06	18
		Drink	35	2	0.06	18
		food/ can eat food	35	2	0.06	18
56	horse	Clip clop	40	2	0.05	22
		riding/ ride on them	40	2	0.05	22
		Tail	40	2	0.05	22
		neigh	40	2	0.05	22
57	house	Tree	39	3	0.08	25
		windows	39	3	0.08	25
		Light. On and off/ lights	39	2	0.05	25
		Toy/ "My got toys in my house"	39	2	0.05	25
58	key	door/ open the door	42	9	0.21	13
		car/ daddy's car/ mummy's car	42	3	0.07	13
		open/ can I open the door	42	3	0.07	13
		lock/ Lock the door	42	2	0.05	13
		Unlock keys/ unlocking	42	2	0.05	13
59	leg	Feet/ foot	41	7	0.17	16
		toe/ toes	41	4	0.10	16
		hands	41	2	0.05	16
		Arm	41	2	0.05	16
		Head	41	2	0.05	16
		knee	41	2	0.05	16
		walk	41	2	0.05	16
60	lion	roar/ they roar	38	9	0.24	17
		claws/ got big claws	38	2	0.05	17
		Baby lion	38	2	0.05	17
		Tail	38	2	0.05	17
		Zoo/ see them in the zoo	38	2	0.05	17
61	lorry/ truck	Wheel/ wheels	38	4	0.11	22
		Digger	38	3	0.08	22
		drive/ DEF: It's something that drives	38	2	0.05	22
62	monkey	banana	35	5	0.14	13
		elephant	35	3	0.09	13
		Swing/ swing in branches	35	3	0.09	13
		cheeky/ cheeky monkey	35	3	0.09	13
		tree/ trees	35	3	0.09	13
		Oo oo aa/ ooo ooo ooo	35	2	0.06	13
63	mouse	cheese/ Eats cheese	36	2	0.06	25
		Run/ running away	36	2	0.06	25
		It squeaks/ goes squeak	36	2	0.06	25
		Squeak	36	2	0.06	25
		tree/ trees	36	2	0.06	25
64	mouth	teeth	30	7	0.23	17
		eat/ eating	30	2	0.07	17
		Gum/ gums	30	2	0.07	17
		Tongue	30	2	0.07	17
		Talking	30	2	0.07	17
65	nappy	Bayb/ Babies	32	5	0.16	18
		Poo/ poop/ We don’t poop	32	4	0.13	18
		bum/ nappies go on your bum	32	2	0.06	18
		Night time/ Nighttime when you wear a nappy	32	2	0.06	18
66	nose	Bogies/ Boogeys	37	3	0.08	18
		Glasses/ glasses(Glasses were on nanny’s nose whilst doing the task)	37	2	0.05	18
		Nostril	37	2	0.05	18
		Tongue	37	2	0.05	18
67	orange	Red	43	3	0.07	22
		orange juice	43	2	0.05	22
		Apple	43	2	0.05	22
		fruit	43	2	0.05	22
		Yellow	43	2	0.05	22
68	park	swing/ swings/ go on the swings	45	7	0.16	23
		slide/ Slide everyday/ Slide on the slide/ slides/ go on the slide	45	7	0.16	23
		Play/ play at the park/ playing	45	4	0.09	23
		roundabout/ go on the roundabout	45	2	0.04	23
		tree/ trees	45	2	0.04	23
69	pasta	Eat/ eat up/ eating	37	7	0.19	23
		sauce/ pasta sauce/ saucy sauce	37	4	0.11	23
		cheese	37	3	0.08	23
		tomato/ tomatoes	37	2	0.05	23
70	peas	eat/ Eating/ We eat them	37	8	0.22	24
71	pen	Draw/ drawing/ DEF: It's something that you draw with	41	6	0.15	16
		Pencil	41	4	0.10	16
		Paper/ Colour on paper	41	3	0.07	16
		Chickens/ Chicken (she said the chicken is in a pen)	41	2	0.05	16
		colouring	41	2	0.05	16
		Crayon	41	2	0.05	16
		write/ writing	41	2	0.05	16
72	phone/ telephone/ mobile	Hello/ say hello	32	3	0.09	20
		Ring/ ring ring	32	3	0.09	20
		Watching/ watch	32	2	0.06	20
73	pig	‘Oink’/ Oink Oink	42	6	0.14	22
		Pink	42	3	0.07	22
		Peppa/ Peppa pig	42	2	0.05	22
		Farm	42	2	0.05	22
		House/ houses	42	2	0.05	22
74	plane/ aeroplane	Fly	35	4	0.11	19
		Sky	35	4	0.11	19
		people	35	2	0.06	19
		Sit down/ People sit down	35	2	0.06	19
75	plate	Eat/ eating/ We eat food off the plate	34	6	0.18	20
		Food	34	5	0.15	20
		lunch	34	2	0.06	20
		spoon	34	2	0.06	20
		washing up	34	2	0.06	20
76	pushchair/ buggy	push/ People push/ pushing	31	4	0.13	16
		Baby/ babies	31	3	0.10	16
		pram	31	2	0.06	16
		wheels	31	2	0.06	16
		Chair/ big chair	31	2	0.06	16
		raincover	31	2	0.06	16
77	pyjamas/ Pjs/ jim jams	Bed/ Sleep in bed	38	8	0.21	19
		Sleep	38	2	0.05	19
		Bath/ bath (bedtime routine)	38	2	0.05	19
		Bedtime/ At bed time	38	2	0.05	19
		nice and warm/ warm	38	2	0.05	19
78	rabbit	Peter Rabbit/ Peter/ Peter (loves Peter Rabbit)	39	4	0.10	21
		Benjamin (loves Peter Rabbit)/ Benjamin bunny	39	3	0.08	21
		carrot/ carrots	39	3	0.08	21
		Hop	39	3	0.08	21
		Rabbit ears/ big ears	39	2	0.05	21
		tail	39	2	0.05	21
79	settee/ sofa/ couch	pillow/ pillows	35	4	0.11	17
		cushion	35	3	0.09	17
		Tellie/ TV/ watching tv	35	3	0.09	17
		Blanket	35	2	0.06	17
		cuddles	35	2	0.06	17
		sit	35	2	0.06	17
80	sheep	Cow/ cows	36	5	0.14	17
		Lamb/ lambs	36	5	0.14	17
		Grass	36	4	0.11	17
		horse/ horseys	36	2	0.06	17
81	shoes	walk/ Go for a walk	40	4	0.10	20
		Feet	40	2	0.05	20
		Put on/ Shoes on	40	2	0.05	20
82	slide	"weeeeeee"/ ‘weeee’	33	4	0.12	16
		Swing	33	3	0.09	16
		down	33	2	0.06	16
		Ladder	33	2	0.06	16
		Park/ In the park	33	2	0.06	16
83	sock	feet/ foot/ put them on your feet	35	7	0.20	16
		smelly/ smelly sock	35	5	0.14	16
		Dressed/ get dressed	35	3	0.09	16
		on/ put on/ on to play	35	3	0.09	16
		Toes	35	2	0.06	16
84	spoon	Fork	34	3	0.09	21
		Yoghurt	34	3	0.09	21
		Breakfast	34	2	0.06	21
		Eating	34	2	0.06	21
		Knife	34	2	0.06	21
		Bowl	34	2	0.06	21
85	stairs	Upstairs/ Daddy do work upstairs	30	5	0.17	20
		climb/ climbing	30	3	0.10	20
		Shoes (I usually put our shoes on the stairs to go upstairs)/ shoes. Muddy shoes up the stairs	30	2	0.07	20
86	swing	park/ play park/ They are at the park but we can’t go to the park because of the germs.	34	6	0.18	20
		sit on/ sitting/ We Sit on them	34	4	0.12	20
		fun	34	2	0.06	20
		hand/ Hand in the air	34	2	0.06	20
87	table	Chair/ chairs	30	4	0.13	19
		eat/ eating	30	4	0.13	19
		breakfast	30	2	0.07	19
		Food	30	2	0.07	19
88	teddy/ teddy bear	cuddle/ cuddling/ cuddly/ cudddles	29	7	0.24	15
		Bed/ going to bed/ Into bed	29	5	0.17	15
		Sleep/ sleeping	29	2	0.07	15
89	television/ telly/ TV	watch	36	4	0.11	20
90	tiger	Lion/ Um.. lion!/ Yes…lion! (makes lion noises and pretends to be a lion)	39	5	0.13	23
		"Roar"/ raaarrgh/ rahhhhh/ rawr	39	4	0.10	23
		Stripes/ stripy/ stripey	39	3	0.08	23
		dinosaur	39	2	0.05	23
		orange/ orange lines	39	2	0.05	23
		Sharp teeth	39	2	0.05	23
91	toast	jam	50	6	0.12	27
		Eat	50	4	0.08	27
		Bread	50	4	0.08	27
		butter	50	3	0.06	27
		Toaster	50	3	0.06	27
		breakfast	50	2	0.04	27
		honey/ And honey	50	2	0.04	27
		peanut butter	50	2	0.04	27
92	toe	feet/ foot	41	4	0.10	21
		nail/ nails	41	2	0.05	21
		Shoe/ shoes	41	2	0.05	21
		sock	41	2	0.05	21
93	tooth/ teeth	brush/ brushing/ You brush you teeth very slowly	43	7	0.16	15
		toothbrush/ use a toothbrush/ Unicorn rainbow brush	43	6	0.14	15
		Toothpaste/ use toothpaste/ Pink toothpaste	43	6	0.14	15
		mouth	43	3	0.07	15
		Water	43	2	0.05	15
94	toothbrush	Toothpaste/ paste	35	9	0.26	14
		Clean (When I clean her teeth we talk about teeth being shiny and clean)/ Teeth clean/ DEF: It's something I clean my teeth with (action).	35	3	0.09	14
		teeth/ Brush teeth	35	3	0.09	14
95	towel	bathroom	36	2	0.06	16
		green	36	2	0.06	16
		swimming	36	2	0.06	16
		Dry	36	3	0.08	16
		bath	36	5	0.14	16
96	toy	play/ Play with toys/ To play with/ We play with the toys	37	5	0.14	25
		dinosaur	37	2	0.05	25
		Game/ play game	37	2	0.05	25
		Teddy bear	37	2	0.05	25
		train/ trains	37	2	0.05	25
97	train	Choo choo	31	4	0.13	19
		Thomas	31	3	0.10	19
		track/ tracks/ train track	31	3	0.10	19
		Santa	31	2	0.06	19
		wheels	31	2	0.06	19
98	tree	leaf/ leaves	32	6	0.19	18
		bird/ birds	32	4	0.13	18
		apples/ Picking apples	32	2	0.06	18
		squirrels	32	2	0.06	18
99	trousers	leg/ legs	34	4	0.12	15
		wear/ wear some	34	2	0.06	15
		Jeans	34	2	0.06	15
		pants	34	2	0.06	15
		put it on/ Put them on when we get dressed	34	2	0.06	15
		socks	34	2	0.06	15
100	window	Door	44	4	0.09	24
		Clean/ cleaning	44	3	0.07	24
		Flowers/ Flowers too	44	3	0.07	24
		Glass	44	3	0.07	24
		Open	44	2	0.05	24
		raining/ rain	44	2	0.05	24
		Shut	44	2	0.05	24
		curtain/ Curtains and blinds	44	2	0.05	24

**Table 7:** Experiment 1. First responses produced by 2+ children in the parentally administered WA task (ordered alphabetically by cue word)

	Cue	Response	No. participants receiving cue (G)	No. participants producing response (P)	Associative strength: FSG (P/G)	Idiosyncratic responses
1	apple	Pear	13	2	0.15	5
		eat/eat it/you can eat it	13	6	0.46	5
2	arm	hand/hands	17	2	0.12	8
		leg/legs	17	4	0.24	8
3	ball	kick/kicking	14	5	0.36	8
		football	14	2	0.14	8
4	balloon	holding/we hold them/holding a balloon	15	3	0.2	9
		pop	15	2	0.13	9
5	banana	eat it/eat/eating	14	5	0.36	9
6	bath/bathtub	wash/to wash ourselves/wash hair	15	3	0.2	10
7	bed	teddy/teddy bear/lambie (teddy)/cuddle up with teddies	15	3	0.2	7
		sleep/to sleep	15	4	0.27	7
8	bee	flower/flowers	14	2	0.14	9
		honey	14	3	0.21	9
9	bib	a baby/baby	17	3	0.18	11
		food	17	3	0.18	11
		no (she doesn’t wear a bib anymore, her decision. this ‘no’ is her saying no to wearing a bib.)	17	2	0.12	11
10	bicycle	riding/ride	16	2	0.13	13
11	bin	rubbish/rubbish in the bin/put rubbish in it	14	6	0.43	6
12	bird	feathers/feather	15	2	0.13	10
		nest	15	3	0.2	10
13	biscuit	eat/eat! (shouts excitedly)/eating	14	3	0.21	9
		chocolate/chocolate biscuit	14	2	0.14	9
14	boat	water/in the water/we was on a boat on water/swim in water	15	4	0.27	7
		sailing/sail	15	2	0.13	7
15	book	read/reading/read story	14	5	0.36	5
		pages	14	2	0.14	5
16	boots	puddle/puddles/jumping in muddy puddles/muddy puddles/splashing in muddy puddles	14	2	0.14	7
		walking/walk	14	2	0.14	7
		wellies	14	2	0.14	7
17	bottle	water/water bottle	15	3	0.2	8
		milk	15	2	0.13	8
		lid	15	2	0.13	8
18	bowl	breakfast	14	2	0.14	9
19	box	make (makes models from boxes)/make something/making	17	3	0.18	10
		put stuff in it/stuff	17	2	0.12	10
20	bread	eat/i eat it	16	2	0.13	10
		toast	16	2	0.13	10
21	brush	hair/brush everyone hair/brush hair/sophie’s long hair/hairbrush	15	6	0.4	8
		teeth/brush your teeth	15	3	0.2	8
22	bubbles	pop/def: they're something that pop. pop the bubbles/	18	5	0.28	10
		blowing/blow/blow bubbles	18	2	0.11	10
23	bus	big/big bus	14	2	0.14	11
		we go on the bus/take us somewhere we like to go	14	2	0.14	11
24	butterfly	flying/fly away/flies	14	3	0.21	8
		wings/yellow wings/yellow and blue wings	14	3	0.21	8
25	cake	birthday	15	2	0.13	10
		eat/eat it/after you make it you eat the cake	15	3	0.2	10
26	car	wheels/wheels to bump	16	3	0.19	8
27	carrots	eat/eat them/eating	14	3	0.21	8
		crunchy/crunch crunch	14	2	0.14	8
28	cat	dog	19	6	0.32	10
29	cereal	eat cereal/eat	16	2	0.13	14
30	chair	sitting/sit/sit down	13	4	0.31	8
31	cheese	eat it/eat/eating/def: easy- it's something that you eat and it's so squeezey	15	5	0.33	8
32	chicken	eggs/egg	15	3	0.2	11
		eat/eating/we eat it	15	2	0.13	11
33	coat	cold	14	2	0.14	10
		jacket	14	2	0.14	10
34	cot	baby/babies/baby sleeps	13	3	0.23	7
35	cow	milk/ae some milk	14	3	0.21	7
		moo	14	2	0.14	7
		pig	14	2	0.14	7
36	cup	drink/drink it/ daddy drinking	15	2	0.13	5
		tea	15	2	0.13	5
		milk	15	2	0.13	5
		water	15	3	0.2	5
37	dog	woof/woof woof	15	3	0.2	10
		cat/kitty cat	15	5	0.33	10
38	doll	boy	17	2	0.12	11
39	door	open/open it	16	3	0.19	12
40	duck	water	13	3	0.23	6
		quack	13	5	0.38	6
41	ear	listen/listening ears	14	2	0.14	8
42	elephant	trunk	15	4	0.27	5
43	eye	spy/I spy	15	2	0.13	10
44	feet	toes	14	2	0.14	11
45	finger	hand/hands	16	5	0.31	7
46	fish	swimming/swim	14	2	0.14	8
		water/lives in water	14	4	0.29	8
		fingers/fish finger/eat fish fingers	14	2	0.14	8
47	flower	bee/bees/buzzy bee	15	4	0.27	7
		petals	15	2	0.13	7
48	fork	spoon	18	5	0.28	5
		knife	18	3	0.17	5
		to eat/eat	18	4	0.22	5
49	frog	frog says ribbit/ribbit	16	2	0.13	12
		water	16	3	0.19	12
50	hair	cut your hair/cutting	15	2	0.13	7
		brush	15	2	0.13	7
		head	15	3	0.2	7
51	hand	foot	14	2	0.14	6
		fingers/finger	14	3	0.21	6
52	hat	head/it goes on your head	14	2	0.14	9
		wear it/we can wear hat	14	2	0.14	9
53	head	hair/hair on	15	6	0.4	7
54	key	lock/lock the door	17	2	0.12	6
		door	17	7	0.41	6
55	leg	foot/feet	18	5	0.28	10
56	lion	roar/they roar/raah	13	9	0.69	3
57	lorry/ truck	drive/def: it's something that drives	15	2	0.13	10
		wheel/wheels	15	2	0.13	10
58	monkey	elephant	14	3	0.21	7
		swing/swing in branches	14	2	0.14	7
		banana	14	3	0.21	7
59	mouse	squeak/goes squeak/it squeaks	15	3	0.2	12
60	mouth	hair off/hair	15	2	0.13	9
		teeth	15	2	0.13	9
		tongue/points to tongue	15	3	0.2	9
61	nappy	baby/babies	16	3	0.19	12
		put nappy on/put on people	16	2	0.13	12
62	nose	bogies/boogeys	15	2	0.13	11
63	orange	fruit	16	2	0.13	10
64	park	swings/swing/go on the swings	17	3	0.18	12
		play/play at a park/playing	17	3	0.18	12
65	pasta	eat/eating/eat up	16	5	0.31	9
		cheese/cream cheese	16	2	0.13	9
		dinner	16	2	0.13	9
66	peas	eat/we eat them/eating	13	3	0.23	
67	pen	draw/drawing/ it's something that you draw with	16	4	0.25	10
		write/writing	16	2	0.13	10
68	phone/ telephone/ mobile	ring/ring ring	14	2	0.14	8
69	pig	oink/oink oink	16	3	0.19	10
		pink	16	2	0.13	10
70	plane	people	16	2	0.13	10
		sky	16	3	0.19	10
71	plate	lunch	14	2	0.14	7
		food/we eat food off the plate	14	5	0.36	7
		eating/eat	14	3	0.21	7
72	pushchair/buggy	push/pushing/people push	14	2	0.14	6
		baby/babies	14	2	0.14	6
		pram/maia goes in pram	14	3	0.21	6
		wheels	14	2	0.14	6
73	pyjamas/Pjs/jim jams	bed	16	3	0.19	10
		bedtime/at bed time	16	2	0.13	10
74	rabbit	peter rabbit/peter (loves peter rabbit)	15	4	0.27	8
		rabbit ears/big ears	15	2	0.13	8
		hop	15	2	0.13	8
75	settee/sofa/couch	cushion	14	3	0.21	8
		pillows/pillow	14	3	0.21	8
76	sheep	cow/cows	18	5	0.28	6
		lambs/lamb	18	4	0.22	6
		grass	18	2	0.11	6
77	shoes	feet	14	2	0.14	10
78	slide	park/in the park	16	2	0.13	10
		ladder	16	2	0.13	10
		swing	16	2	0.13	10
		down	16	2	0.13	10
79	sock	foot/feet/put them on your feet	13	5	0.38	6
		smelly/smelly sock	13	4	0.31	6
80	spoon	breakfast	15	2	0.13	8
		fork	15	3	0.2	8
81	stairs	upstairs	14	3	0.21	9
		climb/climbing	14	3	0.21	9
82	swing	fun	14	2	0.14	8
		we sit on them/sit on/sit/sitting	14	3	0.21	8
		park/play park/they are at the park but we can’t go to the park because of the germs.	14	2	0.14	8
83	table	eat/eating/eat dinner	14	4	0.29	7
		chair/chairs	14	3	0.21	7
84	teddy/teddy bear	cuddle/cuddling/cuddles/cuddly	14	4	0.29	7
		bed/going to bed/into bed/bedtime	14	4	0.29	7
85	television/ telly/TV	watch	14	3	0.21	8
86	tiger	rawr/"roar" (nb. he leapt up with sound affects)/rahhhhh/raaarrgh	14	3	0.21	8
		stripes/stripy/stripey	14	2	0.14	8
87	toast	breakfast/mommy breakfast	19	2	0.11	10
		eat	19	3	0.16	10
		bread	19	4	0.21	10
		jam	19	2	0.11	10
88	toe	foot/feet	17	4	0.24	10
89	tooth/ teeth	toothbrush/use a toothbrush	18	4	0.22	8
		brush/brushing/you brush you teeth very slowly/tiny little brushs (she puts two fingers together)/unicorn rainbow brush	18	6	0.33	8
90	toothbrush	mouth/brushing my mouth	12	2	0.17	5
		teeth/teeth clean/brush teeth	12	3	0.25	5
		toothpaste/paste	12	4	0.33	5
91	towel	bath/bath (he'd just got out the bath)/bath time	14	5	0.36	6
		bathroom	14	2	0.14	6
92	toy	play/play time/to play with/we play with the toys	14	4	0.29	10
93	train	choo choo	14	3	0.21	8
		tracks/track/train track	14	3	0.21	8
94	tree	leaf/leaves	15	4	0.27	8
		birds/bird	15	2	0.13	8
		apples/picking apples	15	2	0.13	8
95	trousers	put it on/put them on when we get dressed	14	2	0.14	6
		leg/legs	14	3	0.21	6
		wear some/wear	14	2	0.14	6
		jeans/red jeans	14	2	0.14	6
96	window	raining/rain	18	2	0.11	12
		door	18	2	0.11	12

**Table 8:** Experiment 1. First responses (nouns) produced by 2+ children in the parentally administered WA task

	Cue	Response	No. participants receiving cue (G)	No. participants producing response (P)	Associative strength: FSG (P/G)	Idiosyncratic responses
1	bin	rubbish/rubbish in the bin/put rubbish in it	14	6	0.43	6
2	key	door	17	7	0.41	6
3	brush	hair/brush everyone hair/brush hair/Sophie’s long hair/hairbrush	15	6	0.4	8
4	head	hair/hair on	15	6	0.4	7
5	sock	foot/feet/put them on your feet	13	5	0.38	6
6	plate	food/we eat food off the plate	14	5	0.36	7
7	towel	bath/bath (he'd just got out the bath)/bath time	14	5	0.36	6
8	dog	cat/kitty cat	15	5	0.33	10
9	toothbrush	toothpaste/paste	12	4	0.33	5
10	cat	dog	19	6	0.32	10
11	finger	hand/hands	16	5	0.31	7
12	fish	water/lives in water	14	4	0.29	8
13	teddy/ teddy bear	bed/going to bed/into bed/bedtime	14	4	0.29	7
14	fork	spoon	18	5	0.28	5
15	leg	foot/feet	18	5	0.28	10
16	sheep	cow/cows	18	5	0.28	6
17	boat	water/in the water/we was on a boat on water/swim in water	15	4	0.27	7
18	elephant	trunk	15	4	0.27	5
19	flower	bee/bees/buzzy bee	15	4	0.27	7
20	rabbit	peter rabbit/peter (loves peter rabbit)	15	4	0.27	8
21	tree	leaf/leaves	15	4	0.27	8
22	arm	leg/legs	17	4	0.24	8
23	toe	foot/feet	17	4	0.24	10
24	cot	baby/babies/baby sleeps	13	3	0.23	7
25	duck	water	13	3	0.23	6
26	sheep	lambs/lamb	18	4	0.22	6
27	tooth/ teeth	toothbrush/use a toothbrush	18	4	0.22	8
28	bee	honey	14	3	0.21	9
29	butterfly	wings/yellow wings/yellow and blue wings	14	3	0.21	8
30	cow	milk/ae some milk	14	3	0.21	7
31	hand	fingers/finger	14	3	0.21	6
32	monkey	elephant	14	3	0.21	7
33	monkey	banana	14	3	0.21	7
34	pushchair /buggy	pram/Maia goes in pram	14	3	0.21	6
35	settee /sofa/couch	cushion	14	3	0.21	8
36	settee/ sofa/couch	pillows/pillow	14	3	0.21	8
37	stairs	upstairs	14	3	0.21	9
38	table	chair/chairs	14	3	0.21	7
39	toast	bread	19	4	0.21	10
40	train	tracks/track/train track	14	3	0.21	8
41	trousers	leg/legs	14	3	0.21	6
42	bed	teddy/teddy bear/lambie (teddy)/cuddle up with teddies	15	3	0.2	7
43	bird	nest	15	3	0.2	10
44	bottle	water/water bottle	15	3	0.2	8
45	brush	teeth/brush your teeth	15	3	0.2	8
46	chicken	eggs/egg	15	3	0.2	11
47	cup	water	15	3	0.2	5
48	hair	head	15	3	0.2	7
49	spoon	fork	15	3	0.2	8
50	car	wheels/wheels to bump	16	3	0.19	8
51	frog	water	16	3	0.19	12
52	nappy	baby/babies	16	3	0.19	12
53	plane	sky	16	3	0.19	10
54	pyjamas/Pjs/ jim jams	bed	16	3	0.19	10
55	bib	a baby/baby	17	3	0.18	11
56	bib	food	17	3	0.18	11
57	park	swings/swing/go on the swings	17	3	0.18	12
58	park	play/play at a park/playing	17	3	0.18	12
59	fork	knife	18	3	0.17	5
60	toothbrush	mouth/brushing my mouth	12	2	0.17	5
61	apple	pear	13	2	0.15	5
62	ball	football	14	2	0.14	8
63	bee	flower/flowers	14	2	0.14	9
64	biscuit	chocolate/chocolate biscuit	14	2	0.14	9
65	book	pages	14	2	0.14	5
66	boots	puddle/puddles/jumping in muddy puddles/muddy puddles/splashing in muddy puddles	14	2	0.14	7
67	boots	wellies	14	2	0.14	7
68	bowl	breakfast	14	2	0.14	9
69	coat	jacket	14	2	0.14	10
70	cow	pig	14	2	0.14	7
71	feet	toes	14	2	0.14	11
72	fish	fingers/fish finger/eat fish fingers	14	2	0.14	8
73	hand	foot	14	2	0.14	6
74	hat	head/it goes on your head	14	2	0.14	9
75	plate	lunch	14	2	0.14	7
76	pushchair/ buggy	baby/babies	14	2	0.14	6
77	pushchair/ buggy	wheels	14	2	0.14	6
78	shoes	feet	14	2	0.14	10
79	swing	park/play park/they are at the park but we can’t go to the park because of the germs.	14	2	0.14	8
80	tiger	stripes/stripy/stripey	14	2	0.14	8
81	towel	bathroom	14	2	0.14	6
82	trousers	jeans/red jeans	14	2	0.14	6
83	bird	feathers/feather	15	2	0.13	10
84	boat	sailing/sail	15	2	0.13	7
85	bottle	milk	15	2	0.13	8
86	bottle	lid	15	2	0.13	8
87	bread	toast	16	2	0.13	10
88	cake	birthday	15	2	0.13	10
89	cup	drink/drink it/ daddy drinking	15	2	0.13	5
90	cup	tea	15	2	0.13	5
91	cup	milk	15	2	0.13	5
92	flower	petals	15	2	0.13	7
93	lorry/ truck	wheel/wheels	15	2	0.13	10
94	mouth	hair off/hair	15	2	0.13	9
95	mouth	teeth	15	2	0.13	9
96	nose	bogies/boogeys	15	2	0.13	11
97	orange	fruit	16	2	0.13	10
98	pasta	cheese/cream cheese	16	2	0.13	9
99	pasta	dinner	16	2	0.13	9
100	plane	people	16	2	0.13	10
101	pyjamas/Pjs/ jim jams	bedtime/at bed time	16	2	0.13	10
102	rabbit	rabbit ears/big ears	15	2	0.13	8
103	slide	park/in the park	16	2	0.13	10
104	slide	ladder	16	2	0.13	10
105	slide	swing	16	2	0.13	10
106	spoon	breakfast	15	2	0.13	8
107	tree	birds/bird	15	2	0.13	8
108	tree	apples/picking apples	15	2	0.13	8
109	arm	hand/hands	17	2	0.12	8
110	doll	boy	17	2	0.12	11
111	sheep	grass	18	2	0.11	6
112	toast	breakfast/mommy breakfast	19	2	0.11	10
113	toast	jam	19	2	0.11	10
114	window	raining/rain	18	2	0.11	12
115	window	door	18	2	0.11	12

**Table 9:** Experiment 1. Related responses given by 2+ children (as first responses) in the parentally administered WA task and represented in adult associative norms

Cue	Response	Child FSG	Adult FSG
apple	pear	0.15	0.15
	eat/eat it/you can eat it	0.46	0.01
arm	hand/hands	0.12	0.08
	leg/legs	0.24	0.54
ball	kick/kicking	0.36	0.07
	football	0.14	0.04
balloon	pop	0.13	0.15
bath/bathtub	wash/to wash ourselves/wash hair	0.20	0.02
bed	sleep/to sleep	0.27	0.64
bee	honey	0.21	0.22
bib	a baby/baby	0.18	0.63
bicycle	riding/ride	0.13	0.19
bin	rubbish/rubbish in the bin/put rubbish in it	0.43	0.33
bird	feathers/feather	0.13	0.06
	nest	0.20	0.04
biscuit	eat/eat! (shouts excitedly)/eating	0.21	0.02
	chocolate/chocolate biscuit	0.14	0.08
boat	water/in the water/we was on a boat on water/swim in water	0.27	0.24
	sailing/sail	0.13	0.14
book	read/reading/read story	0.36	0.33
	pages	0.14	0.06
boots	walking/walk	0.14	0.02
bottle	water/water bottle	0.20	0.03
bowl	breakfast	0.14	0.02
bread	eat/i eat it	0.13	0.03
brush	hair/brush everyone hair/brush hair/Sophie’s long hair/hairbrush	0.40	0.32
	teeth/brush your teeth	0.20	0.16
bubbles	pop/def: they're something that pop. pop the bubbles/	0.28	0.02
	blowing/blow/blow bubbles	0.11	0.03
butterfly	flying/fly away/flies	0.21	0.08
	wings/yellow wings/yellow and blue wings	0.21	0.09
cake	birthday	0.13	0.07
	eat/eat it/after you make it you eat the cake	0.20	0.09
car	wheels/wheels to bump	0.19	0.04
carrots	eat/eat them/eating	0.21	0.01
	crunchy/crunch crunch	0.14	0.01
cat	dog	0.32	0.59
cereal	eat cereal/eat	0.13	0.03
chair	sitting/sit/sit down	0.31	0.21
cheese	eat it/eat/eating/def: easy- it's something that you eat and it's so squeezey	0.33	0.02
chicken	eggs/egg	0.20	0.02
	eat/eating/we eat it	0.13	0.02
coat	cold	0.14	0.07
	jacket	0.14	0.15
cot	baby/babies/baby sleeps	0.23	0.64
cow	milk/ae some milk	0.21	0.35
	moo	0.14	0.06
	pig	0.14	0.02
cup	drink/drink it/ daddy drinking	0.13	0.03
	tea	0.13	0.07
	water	0.20	0.06
dog	cat/kitty cat	0.33	0.59
door	open/open it	0.19	0.16
duck	water	0.23	0.02
	quack	0.38	0.11
ear	listen/listening ears	0.14	0.03
elephant	trunk	0.27	0.21
feet	toes	0.14	0.28
finger	hand/hands	0.31	0.24
fish	swimming/swim	0.14	0.08
	water/lives in water	0.29	0.09
	fingers/fish finger/eat fish fingers	0.14	0.04
flower	petals	0.13	0.17
fork	spoon	0.28	0.33
	knife	0.17	0.41
	to eat/eat	0.22	0.05
fish	water	0.19	0.02
hair	cut your hair/cutting	0.13	0.04
	brush	0.13	0.11
	head	0.20	0.03
hand	foot	0.14	0.13
	fingers/finger	0.21	0.23
hat	head/it goes on your head	0.14	0.22
head	hair/hair on	0.40	0.13
key	lock/lock the door	0.12	0.37
	door	0.41	0.19
leg	foot/feet	0.28	0.13
lion	roar/they roar/raah	0.69	0.03
lorry/ truck	drive/def: it's something that drives	0.13	0.02
	wheel/wheels	0.13	0.02
monkey	swing/swing in branches	0.14	0.01
	banana	0.21	0.05
mouth	teeth	0.13	0.18
	tongue/points to tongue	0.20	0.08
orange	fruit	0.13	0.15
park	swings/swing/go on the swings	0.18	0.04
	play/play at a park/playing	0.18	0.02
peas	eat/we eat them/eating	0.23	0.01
pen	write/writing	0.13	0.07
phone/ telephone/ mobile	ring/ring ring	0.14	0.27
pig	oink/oink oink	0.19	0.04
	pink	0.13	0.02
plane	sky	0.19	0.09
plate	food/we eat food off the plate	0.36	0.19
	eating/eat	0.21	0.05
pushchair	baby/babies	0.14	0.07
pyjamas/Pjs/ jim jams	bed	0.19	0.15
	bedtime/at bed time	0.13	0.02
rabbit	peter rabbit/peter (loves peter rabbit)	0.27	0.01
	rabbit ears/big ears	0.13	0.05
	hop	0.13	0.02
settee/sofa/ couch	cushion	0.21	0.05
sheep	cow/cows	0.28	0.07
	lambs/lamb	0.22	0.09
shoes	feet	0.14	0.33
slide	park/in the park	0.13	0.02
	swing	0.13	0.11
	down	0.13	0.09
sock	foot/feet/put them on your feet	0.38	0.17
spoon	fork	0.20	0.50
stairs	upstairs	0.21	0.14
	climb/climbing	0.21	0.23
swing	fun	0.14	0.01
	park/play park/they are at the park but we can’t go to the park because of the germs.	0.14	0.08
table	eat/eating/eat dinner	0.29	0.03
	chair/chairs	0.21	0.76
television/ telly/TV	watch	0.21	0.09
tiger	rawr/"roar" (nb. he leapt up with sound affects)/rahhhhh/raaarrgh	0.21	0.02
	stripes/stripy/stripey	0.14	0.08
toast	breakfast/mommy breakfast	0.11	0.07
	eat	0.16	0.02
	bread	0.21	0.36
	jam	0.11	0.01
toe	foot/feet	0.24	0.58
tooth/ teeth	toothbrush/use a toothbrush	0.22	0.02
	brush/brushing/you brush your teeth very slowly/tiny little brushes (she puts two fingers together)/unicorn rainbow brush	0.33	0.10
toothbrush	mouth/brushing my mouth	0.17	0.02
	teeth/teeth clean/brush teeth	0.25	0.16
	toothpaste/paste	0.33	0.32
towel	bath/bath (he'd just got out the bath)/bath time	0.36	0.05
	bathroom	0.14	0.02
toy	play/play time/to play with/we play with the toys	0.29	0.10
train	choo choo	0.21	0.03
	tracks/track/train track	0.21	0.18
tree	leaf/leaves	0.27	0.18
trousers	leg/legs	0.21	0.01
	wear some/wear	0.14	0.02
	jeans/red jeans	0.14	0.04
window	door	0.11	0.15

**Table 10:** Experiment 1. Related responses given by 2+ children (as first responses) in the parentally-administered WA task, and not represented in adult associative norms (n/d = not documented, n/c = not used as a cue)

Cue	Response	Child FSG	South Florida Norms: Nelson et al. (1998)	Birkbeck Norms: Moss and Older (1996)
banana	eat it/eat/eating	0.36	n/d	n/c
pasta	eat/eating/eat up	0.31	n/d	n/c
sock	smelly/smelly sock	0.31	n/d	n/c
teddy/teddy bear	cuddle/cuddling/cuddles/cuddly	0.29	n/c	n/c
teddy/teddy bear	bed/going to bed/into bed/bedtime	0.29	n/c	n/c
flower	bee/bees/buzzy bee	0.27	n/d	n/d
pen	draw/drawing/ it's something that you draw with	0.25	n/d	n/c
monkey	elephant	0.21	n/d	n/d
pushchair/buggy	pram/maia goes in pram	0.21	n/d	n/c
settee/sofa/couch	pillows/pillow	0.21	n/d	n/d
swing	we sit on them/sit on/sit/sitting	0.21	n/d	n/d
balloon	holding/we hold them/holding a balloon	0.20	n/d	n/d
bed	teddy/teddy bear/lambie (teddy)/cuddle up with teddies	0.20	n/d	n/c
dog	woof/woof woof	0.20	n/d	n/d
mouse	squeak/goes squeak/it squeaks	0.20	n/d	n/d
nappy	baby/babies	0.19	n/c	n/c
bib	food	0.18	n/c	n/d
box	make (makes models from boxes)/make something/making	0.18	n/d	n/d
bee	flower/flowers	0.14	n/d	n/d
boots	puddle/puddles/ jumping in muddy puddles/muddy puddles/splashing in muddy puddles	0.14	n/d	n/d
boots	wellies	0.14	n/d	n/d
bus	big/big bus	0.14	n/d	n/d
bus	we go on the bus/take us somewhere we like to go	0.14	n/d	n/d
hat	wear it/we can wear hat	0.14	n/d	n/d
plate	lunch	0.14	n/d	n/d
pushchair/buggy	push/pushing/people push	0.14	n/d	n/c
pushchair/buggy	wheels	0.14	n/d	n/c
trousers	put it on/put them on when we get dressed	0.14	n/d	n/d
bottle	milk	0.13	n/d	n/c
bottle	lid	0.13	n/d	n/c
bread	toast	0.13	n/d	n/d
cup	milk	0.13	n/d	n/d
eye	spy/I spy	0.13	n/d	n/d
frog	frog says ribbit/ribbit	0.13	n/d	n/d
mouth	hair off/hair	0.13	n/d	n/d
nappy	put nappy on/put on people	0.13	n/c	n/c
nose	bogies/boogeys	0.13	n/d	n/d
pasta	cheese/cream cheese	0.13	n/d	n/c
pasta	dinner	0.13	n/d	n/c
plane	people	0.13	n/d	n/d
slide	ladder	0.13	n/d	n/d
spoon	breakfast	0.13	n/d	n/d
tree	birds/bird	0.13	n/d	n/d
tree	apples/picking apples	0.13	n/d	n/d
bib	no (she doesn’t wear a bib anymore, her decision. this ‘no’ is her saying no to wearing a bib.)	0.12	n/c	n/d
box	put stuff in it/stuff	0.12	n/d	n/d
doll	boy	0.12	n/d	n/d
sheep	grass	0.11	n/d	n/d
window	raining/rain	0.11	n/d	n/c

**Table 11:** Experiment 2. All related responses (first, second and third attempts) produced by 2+ children in the online WA task

	Cue	Response	No. participants receiving cue (G)*3 attempts	No. participants producing response (P)	Associative strength: FSG (P/G)	Idiosyncratic responses
1	bath	we play/ play	36	2	0.06	19
		shower/ i have a shower	36	2	0.06	19
		wash time/ wash yourself in the bath	36	2	0.06	19
		towel	36	2	0.06	19
		not wash my hair/ scrub your hair	36	2	0.06	19
		mummy/ Mummys bath	36	3	0.08	19
		toys/ Toys (bath toys)/ put toys in the bath/ dinosaur (bath toys)	36	6	0.17	19
2	bed	lay on it/ lay in bed	36	2	0.06	15
		teddy	36	2	0.06	15
		pillow/s	36	3	0.08	15
		sleep/ sleeping/ sleep on it	36	3	0.08	15
		toys/ mushroom (soft toy)/ pumpkin (soft toy)	36	5	0.14	15
3	bowl	Poppy - our cat has bowls and he puts the food in them/ cat food	36	2	0.06	13
		spoon	36	2	0.06	13
		you eat food out of your bowl/ eating	36	3	0.08	13
4	brush	combing/ comb	36	2	0.06	20
		pink	36	2	0.06	20
		hair/ we use it for our hair	36	7	0.19	20
5	cereal	milk	36	2	0.06	17
		EAT/ eating	36	2	0.06	17
		porridge	36	2	0.06	17
		breakfast	36	4	0.11	17
6	chair	mummy/ mum	36	2	0.06	11
		table	36	2	0.06	11
		sit on it/ sit	36	3	0.08	11
7	door	handle/door handle	36	2	0.06	17
		close the door/ close	36	2	0.06	17
		outside	36	2	0.06	17
		shut/ shutting	36	3	0.08	17
		opening/ open door/ open/ open and shut them	36	4	0.11	17
8	finger	wiggle your finger/ wiggly worms	36	2	0.06	14
		hand/ hands/ red ouchie on my hand	36	3	0.08	14
9	foot	toes	36	3	0.08	14
		socks on it/ socks	36	3	0.08	14
		shoes/ get you shoes on	36	3	0.08	14
10	hair	wash it with soap/ wash hair and go to bed/ wash your hair	36	3	0.08	13
		brush your hair/ brush/ brushing hair in the bath/ brush it/ brush hair	36	5	0.14	13
11	hand	fingers/ finger	36	2	0.06	14
		Wash/ wash your hands	36	2	0.06	14
12	head	shoulders/ shoulders kneesand toes	36	2	0.06	21
		ears/ ears on your head	36	2	0.06	21
		eyes	36	2	0.06	21
		feet	36	2	0.06	21
		hair/ it has hair/ hair on your head/ cradle cap in my hair	36	4	0.11	21
13	key	lock the car/ car	36	2	0.06	19
		lock/ lock things with a key/ locking us in	36	4	0.11	19
		lock it up with a door/ lock the door/ door/ a door/	36	4	0.11	19
14	park	we play/ play in the mud	36	2	0.06	20
		slide	36	3	0.08	20
		swing/ swings in the park/ we swing/ swings	36	6	0.17	20
15	sock	smelly/ smelly welly	36	2	0.06	15
		pointed to foot	36	3	0.08	15
		wear them on our feet/ pointed to foot/ put on foot/ you put your sock on your feet	36	4	0.11	15
16	swing	wee-weeeee/ weee	36	2	0.06	11
		sit on it	36	2	0.06	11
		up	36	2	0.06	11
		Down	36	2	0.06	11
		push/ push up high/ push it	36	3	0.08	11
		at the park/ park/ park swing	36	4	0.11	11
17	table	dinner and tea/ tea	36	2	0.06	13
		breakfast	36	3	0.08	13
		food/ dinner with food	36	3	0.08	13
		Eat/ eating at the table/ eat/ we eat on it/ we eat pancakes there	36	5	0.14	13
18	teddy	bed/ take them to bed	36	2	0.06	17
		Cuddle/ cuddly unicorn/ cuddling/ cuddle them	36	4	0.11	17
19	tooth	toothpaste	36	2	0.06	17
		bite with them/ indicated biting	36	2	0.06	17
		mummy/ mum	36	2	0.06	17
		brush/ brushing your teeth/ brushing/ Brushing teeth/ brush them	36	5	0.14	17
20	towel	Washing	36	2	0.06	10
		for my face/ face	36	2	0.06	10
		drying off/ dry/ dry hands/ drying/ dry the cat	36	6	0.17	10

**Table 12:** Experiment 2. Associative strength for word pairs from online child data (first responses) and represented in adult norms

	Cue	Response	Child FSG	Adult FSG
1	bed	pillow/s	0.17	0.02
2	bed	sleep/ sleeping/ sleep on it	0.17	0.64
3	brush	hair/ we use it for our hair	0.42	0.32
4	cereal	EAT/ eating	0.17	0.03
5	cereal	breakfast	0.25	0.44
6	chair	sit on it/ sit	0.25	0.21
7	door	close the door/ close	0.17	0.03
8	foot	toes	0.25	0.28
9	hair	brush your hair/ brush/ brushing hair in the bath/ brush it/ brush hair	0.25	0.11
10	head	shoulders/ shoulders kneesand toes	0.17	0.05
11	key	lock/ lock things with a key/ locking us in	0.25	0.37
12	park	swing/ swings in the park/ we swing/ swings	0.33	0.04
13	sock	wear them on our feet/ pointed to foot/ put on foot/ you put your sock on your feet	0.17	0.17
14	swing	at the park/ park/ park swing	0.17	0.07
15	table	Eat/ eating at the table/ eat/ we eat on it/ we eat pancakes there	0.25	0.03
16	tooth	brush/ brushing your teeth/ brushing/ Brushing teeth/ brush them	0.17	0.12
17	towel	drying off/ dry/ dry hands/ drying/ dry the cat	0.33	0.28

**Table 13:** Experiment 2. Imageable noun-noun, cue-response word pairs in the online WA task (first responses in bold)

	Cue	Response	Associative strength: FSG (P/G)
1	bath	shower/ i have a shower	0.06
2	bath	towel	0.06
3	bath	toys/ Toys (bath toys)/ put toys in the bath/ dinosaur (bath toys)	0.17
4	bed	teddy	0.06
**5**	**bed**	**pillow/s**	**0.08**
6	bed	toys/ mushroom (soft toy)/ pumpkin (soft toy)	0.14
7	bowl	spoon	0.06
8	brush	combing/ comb	0.06
**9**	**brush**	**hair/ we use it for our hair**	**0.19**
10	cereal	milk	0.06
11	cereal	porridge	0.06
12	chair	table	0.06
13	door	handle/door handle	0.06
14	finger	hand/ hands/ red ouchie on my hand	0.08
**15**	**foot**	**toes**	**0.08**
16	foot	socks on it/ socks	0.08
17	foot	shoes/ get you shoes on	0.08
**18**	**hair**	**brush your hair/ brush/ brushing hair in the bath/ brush it/ brush hair**	**0.14**
19	hand	fingers/ finger	0.06
**20**	**head**	**shoulders/ shoulders kneesand toes**	**0.06**
21	head	ears/ ears on your head	0.06
22	head	eyes	0.06
23	head	feet	0.06
24	head	hair/ it has hair/ hair on your head/ cradle cap in my hair	0.11
25	key	lock the car/ car	0.06
26	key	lock it up with a door/ lock the door/ door/ a door/	0.11
27	park	slide	0.08
**28**	**park**	**swing/ swings in the park/ we swing/ swings**	**0.17**
**29**	**sock**	**wear them on our feet/ pointed to foot/ put on foot/ you put your sock on your feet**	**0.11**
**30**	**swing**	**at the park/ park/ park swing**	**0.11**
31	teddy	bed/ take them to bed	0.06
32	tooth	toothpaste	0.06
**33**	**tooth**	**brush/ brushing your teeth/ brushing/ Brushing teeth/ brush them**	**0.14**
34	towel	for my face/ face	0.06

**Table 14:** Word associations replicated in Experiments 1 and 2 from all responses

	Cue Word	Responses repeated by 2+ participants	FSG_Online version	FSG_Parent version	FSG_Birkbeck Norms: Moss and Older (1996)	FSG_South Florida Norms: Nelson et al. (1998)
1	brush	hair/ we use if for our hair	0.188	0.190	0.200	0.440
2	hair	brush/ brush it/ brush your hair/ brushing hair in the bath	0.182	0.110	0.021	0.207
3	swing	park/ at the park/ park swing	0.174	0.180	0.067	0.101
4	park	swing/ swings/ swing in the park/ we swing	0.171	0.160	0.021	0.061
5	bath	toys/ put toys in the bath/ dinosaur (bath toys)/ Toys (bath toys)	0.171	0.130	not documented	not documented
6	tooth	brush	0.152	0.160	0.115	0.123
7	door	open/ open door/ open and shut them/ opening	0.148	0.150	0.146	0.183
8	key	door/ lock it up with a door/ lock the door	0.147	0.210	0.156	0.218
9	table	eat/ eating at the table/ we eat on it	0.143	0.130	not a cue	0.026
10	towel	dry/ drying/ drying off	0.143	0.080	not a cue	0.284
11	sock	foot/feet: put on foot/ you put your sock on your feet/ wear them on our feet	0.138	0.200	not a cue	0.172
12	foot	toes	0.125	0.110	0.085	0.466
13	foot	socks. socks on it	0.125	0.050	0.043	not documented
14	cereal	breakfast	0.121	0.050	0.548	0.333
15	key	lock/ lock things with a key/ locking us in	0.118	0.050	0.489	0.255
16	head	hair/ hair on your head/ it has hair/ cradle cap in my hair	0.114	0.220	0.064	0.186
17	door	shut/ shutting	0.111	0.060	0.062	not documented
18	table	breakfast	0.107	0.070	not a cue	not documented
19	teddy	cuddle/ cuddle them/ cuddling	0.100	0.240	not a cue	not a cue
20	hand	finger/ fingers	0.095	0.100	0.095	0.358
21	chair	sit/ sit on it	0.094	0.110	not a cue	0.212
22	swing	sit on it	0.087	0.120	not documented	not documented
23	park	slide	0.086	0.160	not documented	not documented
24	bed	sleep	0.086	0.150	not a cue	0.638
25	foot	shoes	0.083	0.050	0.192	0.108
26	finger	hand	0.074	0.190	0.125	0.268
27	sock	smelly/ smelly welly	0.069	0.140	not a cue	not documented
28	teddy	bed/ take them to bed	0.067	0.170	not a cue	not a cue
29	bowl	food/ you eat food out of your bowl	0.067	0.070	not documented	0.017
30	chair	table	0.063	0.050	not a cue	0.314
31	tooth	toothpaste	0.061	0.140	0.019	0.058
32	cereal	milk	0.061	0.130	not documented	0.204
33	cereal	eat/ eating	0.061	0.080	not documented	0.031
34	key	car/ lock the car	0.059	0.070	not documented	0.115
35	bed	teddy	0.057	0.130	not a cue	not documented
36	bath	wash time/ wash yourself in the bath	0.057	0.080	not documented	0.024
37	head	ears/ ears on your head	0.057	0.060	not documented	not documented
38	head	eyes	0.057	0.060	not documented	not documented

**Table 15:** Word associations replicated in Experiments 1 and 2 as first responses

	Cue	Response	Experiment 1 (parent) FSG	Experiment 2 (online) FSG	FSG_Birkbeck Norms: Moss and Older (1996)	FSG_South Florida Norms: Nelson et al. (1998)
1	bed	sleep	0.27	0.17	not a cue	0.64
2	brush	hair	0.4	0.42	0.20	0.44
3	cereal	eat	0.13	0.17	not documented	0.03
4	chair	sit	0.31	0.25	not a cue	0.21
5	foot	toes	0.14	0.25	0.09	0.47
6	hair	brush	0.13	0.25	0.02	0.21
7	key	lock	0.12	0.25	0.49	0.26
8	park	swing	0.18	0.33	0.02	0.06
9	sock	foot/feet	0.38	0.17	not a cue	0.17
10	swing	park	0.14	0.17	0.07	not a cue
11	table	eat	0.29	0.25	not a cue	0.03
12	teddy	cuddle	0.29	0.25	not a cue	not a cue
13	tooth	brush	0.33	0.17	0.12	0.12

**Table 16:** Experiment 3. Stimuli list for the online semantic priming study on 3-year-olds

	Association Type	Prime	Target	FSG_child Expriment 1	FSG_child Experiment 2	FSG_adult Birkbeck Norms: Moss and Older (1996)	FSG_adult South Florida Norms: Nelson et al. (1998)	Infant study using this WA pair	Distractor
1	Adult+Child	chair	table	0.05	0.06	n/c	0.31	Arias-Trejo & Plunkett (2009); Floccia et al. (2020); Jardak & Byers-Heinlein (2018)*; Singh (2014)*	hand
2	Adult+Child	key	door	0.21	0.14	0.16	0.22		toys
3	Adult+Child	finger	hand	0.19	0.06	0.13	0.27	Floccia et al. (2020)	bib
4	Adult+Child	sock	foot	0.20	0.08	n/c	0.17		mouse
5	UniqueAdult	nappy	bib	n/a	n/a	n/c	n/c	Arias-Trejo & Plunkett (2009); Floccia et al. (2020)	puddle
6	UniqueAdult	elephant	mouse	n/a	n/a	0.07	0.09	Arias-Trejo & Plunkett (2009); Floccia et al. (2020); Singh (2014)*; Styles & Plunkett (2009)	foot
7	UniqueAdult	plate	cup	n/a	n/a	0.24	0.05	Arias-Trejo & Plunkett (2009); Singh (2014)*; Styles & Plunkett (2009)	teddy
8	UniqueAdult	apple	banana	n/a	n/a	0.02	0.02	Arias-Trejo & Plunkett (2009); Floccia et al. (2020); Jardak & Byers-Heinlein (2018)*; Singh (2014)*; Styles & Plunkett (2009)	swing
9	UniqueChild	park	swing	0.16	0.17	0.02	0.06		banana
10	UniqueChild	bed	teddy	0.13	0.06	n/c	n/a		cup
11	UniqueChild	boots	puddle	0.16	n/c	n/a	n/a		table
12	UniqueChild	bath	toys	0.13	0.17	n/a	n/a		door
13	Unrelated	box	mouth	n/a	n/a	n/a	n/a		train
14	Unrelated	duck	hair	n/a	n/a	n/a	n/a		cheese
15	Unrelated	fish	car	n/a	n/a	n/a	n/a		bread
16	Unrelated	bus	pig	n/a	n/a	n/a	n/a		house
17	Unrelated	frog	plane	n/a	n/a	n/a	n/a		slide
18	Unrelated	bike	shoe	n/a	n/a	n/a	n/a		peas
19	Unrelated	cat	bread	n/a	n/a	n/a	n/a		plane
20	Unrelated	cake	house	n/a	n/a	n/a	n/a		pig
21	Unrelated	boat	peas	n/a	n/a	n/a	n/a		hair
22	Unrelated	cot	slide	n/a	n/a	n/a	n/a		mouth
23	Unrelated	pen	train	n/a	n/a	n/a	n/a		shoe
24	Unrelated	hat	cheese	n/a	n/a	n/a	n/a		car

*bilingual studies

Key n/a no associate noted

n/c not used as a cue

## Data Availability

The stimulus materials for Experiment 1 are available in the appendices. For Experiments 2 and 3, an account must be set up on the Gorilla Experiment Builder website (https://gorilla.sc/), before which a copy of all stimuli and the procedure can be shared by sending an email to nadine.fitzpatrick@plymouth.c.uk. The word association data in the Appendices can be found on https://osf.io/t2f69/

## Footnotes

1 It would not be culturally appropriate to ask about a person’s ethnicity in the UK.

2 In a pilot study, we observed that children as young as 24–30 months were not always able to understand and complete the task. We decided to assign children to age bands across the third year of life, to explore whether age was a factor in a child comprehending and being able to complete the task. We limited recruitment to 20 participants per age bin due to funding constraints, but some parents returned the task later than expected (but before analysis had begun), which is why some bands have more than 20 participants.

3 Paradigmatic responses included a superordinate (e.g. cat–animal), a subordinate (e.g. train–carriage), a synonym (e.g. brush–comb), an antonym (e.g. night–day), or a category coordinate (e.g. elephant–dog). Syntagmatic responses were identified if a word was able to syntactically follow or precede the cue (e.g. train–track) or if thematically close (e.g. bed–story). Both indicated that the response satisfied the conditions of both a paradigmatic and syntagmatic response (e.g. cat–dog).

4 When considering all responses, a total of 432 responses had two or more participants producing the same response for a cue word, with all 100 cue words represented in these responses. The full list of cue words (organised alphabetically) with two or more of the same response and their associative strengths (*M* = 0.09, range = 0.04 to 0.29) can be found in Appendix Table 6.

5 When all responses are considered, this increases the percentage of associated responses in children that are not found in adult norms to 42%.

6 This feature did not work correctly online for all participants, and follow-up email versions of the CDI were sent, but not consistently completed and returned to the experimenter; thus these data are largely missing for the sample.

7 For all responses this was 74%.

8 Looking at all responses, a total of 72 responses were given by two or more participants with all 20 cue words represented in these word combinations. The full list of cue–response word pairs with their corresponding associative strengths (*M* = 0.08, range= 0.06 to 0.19) can be found in Appendix Table 11.

9 This percentage increases to 37% when considering all responses and not just first responses.

10 We did not factor in the number of trials we expected to lose, which was an oversight.

11 Floccia et al. (2020) identified the first 1700 ms as the time period in which differences between conditions are seen in 27-month-olds, using the same priming task type on bilinguals.

12 Experiment 1: 26%, Experiment 2: 32%
